# Analgesic Peptides: From Natural Diversity to Rational Design

**DOI:** 10.3390/molecules29071544

**Published:** 2024-03-29

**Authors:** Katarzyna Gach-Janczak, Monika Biernat, Mariola Kuczer, Anna Adamska-Bartłomiejczyk, Alicja Kluczyk

**Affiliations:** 1 Department of Biomolecular Chemistry, Faculty of Medicine, Medical University of Lodz, Mazowiecka 6/8, 92-215 Lodz, Poland; katarzyna.gach@umed.lodz.pl (K.G.-J.); anna.adamska@umed.lodz.pl (A.A.-B.); 2 Faculty of Chemistry, University of Wroclaw, F. Joliot-Curie 14, 50-383 Wroclaw, Poland; monika.biernat@uwr.edu.pl (M.B.); mariola.kuczer@uwr.edu.pl (M.K.)

**Keywords:** natural antinociceptive peptides, opioids, analgesia, nociceptin/orphanin, peptidomimetics, bioconjugates

## Abstract

Pain affects one-third of the global population and is a significant public health issue. The use of opioid drugs, which are the strongest painkillers, is associated with several side effects, such as tolerance, addiction, overdose, and even death. An increasing demand for novel, safer analgesic agents is a driving force for exploring natural sources of bioactive peptides with antinociceptive activity. Since the G protein-coupled receptors (GPCRs) play a crucial role in pain modulation, the discovery of new peptide ligands for GPCRs is a significant challenge for novel drug development. The aim of this review is to present peptides of human and animal origin with antinociceptive potential and to show the possibilities of their modification, as well as the design of novel structures. The study presents the current knowledge on structure-activity relationship in the design of peptide-based biomimetic compounds, the modification strategies directed at increasing the antinociceptive activity, and improvement of metabolic stability and pharmacodynamic profile. The procedures employed in prolonged drug delivery of emerging compounds are also discussed. The work summarizes the conditions leading to the development of potential morphine replacements.

## 1. Introduction: Opioid Peptides and Their Receptors

Pain treatment was one of humanity’s main problems even in ancient times. The pain-relieving properties of opium, the poppy plant (*Papaver somniferum*) extract, have been known for centuries. Even today, the most effective painkiller in the treatment of severe and chronic pain is morphine, an alkaloid obtained from opium. Morphine acts through opioid receptors, localized in the central nervous system (CNS) and many peripheral tissues [1]. There are three main types of opioid receptors designated µ, δ, and κ (or MOR, DOR, and KOR, respectively). These receptors, called classical opioid receptors, were discovered in the 1970s, and they all mediate analgesia in humans as well as in animal models of pain [2,3]. The strongest antinociceptive effect is associated with the activation of the MOR, which plays the main role in the signal transduction cascades responsible for pain perception. Morphine binds to all three types of opioid receptors, but its affinity to MOR is about 100 times greater than that of DOR and KOR [4]. Unfortunately, morphine treatment, especially chronic, causes serious side effects, including sedation, tolerance and dependence, constipation, respiratory depression, and hypotension [5].

The fourth member of the opioid receptor family is the nociceptin/orphanin FQ (N/OFQ) receptor (NOP), previously referred to as opioid-receptor-like1 (ORL1), identified in 1994 [6]. On the basis of its structural similarity to the classical opioid receptors, the NOP receptor was classified as belonging to the opioid receptor family despite its unique pharmacological profile [7].

Opioid receptors are members of the G protein-coupled receptors (GPCRs) family and are coupled with G_i_/G_o_ proteins. GPCR activation leads to the modulation of various intracellular signaling partners, including adenylyl cyclase, phospholipase C, ion channels, and components of the mitogen-activated protein kinase (MAPK) pathway [8]. GPCR stimulation inhibits the activity of adenylyl cyclase and cAMP production, triggering the modulation of synaptic plasticity, pain processing, and memory and reward processing [9,10]. The opening of inwardly rectifying K^+^ channels and inhibition of voltage-gated dependent Ca^2+^ channels (VDCCs) cause intracellular hyperpolarization of the neuron and a reduction in the release of presynaptic neurotransmitters such as glutamate and substance P, which are vital in the transmission of pain. Thus, the activation of opioid receptors creates a strong analgesic effect [9].

The discovery of opioid receptors resulted in the search for their endogenous ligands, known as opioid peptides [2,11]. In the 1970s, DOR-selective enkephalins [12], KOR-selective dynorphins [13,14], and non-selective β-endorphins [15] were isolated from the mammalian brain. All these peptides have the same *N*-terminal amino acid sequence (Tyr-Gly-Gly-Phe) and free carboxylic group at the *C*-terminus. Peptides with such structure were named “typical” opioid peptides. Much later, in 1997, Zadina and co-workers discovered two tetrapeptides in the bovine brain [16] and human cerebral cortex [17], which showed high affinity and selectivity for MOR. These peptides act through the same opioid receptor as morphine and, therefore, were named endomorphin-1 and endomorphin-2 (Table 1). Endomorphins, unlike other opioid peptides, possess a proline residue in position 2 and have amidated *N*-terminus. Due to these differences, they were named ”atypical” opioid peptides [2].

The endogenous ligand of the NOP receptor was identified in 1995, independently by two groups, and named nociceptin/orphanin FQ (N/OFQ) [18,19]. Nociceptin has no affinity for classical opioid receptors, and MOR and DOR ligands do not bind to the NOP receptor [20]. The main structural difference between nociceptin and other opioid peptides is the presence of Phe instead of Tyr as the *N*-terminal amino acid. The endogenous ligands of all four opioid receptors are presented in Table 1.

Several “atypical” opioid peptides (Table 2) have been obtained from body fluids such as milk and blood, including β-casomorphin [21] and its shorter form, morphiceptin [22,23], that are products of enzymatic digestion of β-casein, while hemorphins originate from the blood protein hemoglobin [24]. Another important group of opioid peptides is compounds isolated from the amphibian skin: MOR-selective dermorfin [25], DOR-selective dermenkephalin [26], and deltorphin I and II [27]. Interestingly, all these peptides from amphibians are characterized by a very high affinity for mammalian opioid receptors (Table 2).

Although all three types of classic opioid receptors are involved in analgesic processes, each activation produces different specific effects. Despite strong antinociceptive activity, activation of MOR may lead to respiratory depression, inhibition of intestinal peristalsis, physical addiction, and euphoria. DOR agonists have reduced addictive potential but also lower antinociceptive efficacy. KOR ligands are responsible for strong dysphoric effects but can also be viewed as potential analgesics only for peripheral use [3,11,28]. The pharmacological profile of the NOP receptor differs from that of classic opioid receptors. The NOP receptor plays important roles in various physiological functions, most importantly in learning and memory, locomotion, and anxiety. Antinociceptive effects mediated via the NOP receptor are complex; activation via N/OFQ was shown to produce either anti- or pro-nociceptive effects, depending on the animal species, dosage, route of administration, as well as the pain model [29,30].

Since the endogenous opioid system is known to play an essential role in pain perception and modulation, opioid receptors and their ligands have been important targets in medicinal chemistry. Numerous opioid peptides and their synthetic analogs have been studied extensively in order to determine their structure–activity relationship (SAR) and also in the hope of finding structural leads for novel analgesics safer than morphine [11,31,32,33].

The opioid system, discussed in detail above, has an indisputable and long history in the treatment of pain [8]. However, in addition to the opioidergic system, there are several other systems, such as neurotensin, cannabinoid, neurokinin, and melanocortin, as well as varieties of neurotransmitters involved in the modulation of pain perception. The major types of pain-associated neurotransmitters (inflammatory mediators: prostaglandins PGE_2_ and PGI_2_, leukotriene B_4_ (LTB_4_), nerve growth factor (NGF), proton, bradykinin, ATP, adenosine, substance P, neurokinins A and B, 5-hydroxytryptamine (5-HT), histamine, glutamate, norepinephrine and NO; non-inflammatory mediators: calcitonin gene-related peptide (CGRP), GABA, glycine and cannabinoids), their cognate receptors (either pre- or post-synaptic) and eventual pharmacological effects on pain regulation are discussed in excellent review by Yam et al. [34].

Due to their evolutionary role, various natural compounds are regular ligands of receptors involved in physiological functions, including pain modulation, and, at the same time, they are inspirations for the design and development of novel bioactive compounds. Among them, nature-derived peptides, mostly GPCR ligands, are frequently studied because of their selectivity and the accumulated knowledge on peptide design and synthesis for specific purposes. The well-known metabolism and vast possibilities for derivatization make peptides one of the favorite starting points in the search for drug candidates [35].

This review aims to present natural sources of potential analgesic peptides and methods of modification, as well as the design of novel structures with improved bioavailability and pharmacodynamic properties. We will discuss antinociceptive peptides from the animal kingdom and the strategies employed in the transformation of their sequences into more drug-like molecules.

Pain affects one-third of the global population, and it is a public health issue. Currently, opioid drugs are associated with several disorders, such as tolerance, addiction, overdose, and sometimes death [36]. Problems related to the use of opioid analgesics in patients have prompted scientists to search for alternative drugs with new structures and improved properties.

## 2. Antinociceptive Peptides of Animal Origin

The exploration of alternative therapeutic options has led researchers to delve into the world of animal peptides [37,38,39]. Many creatures in the animal kingdom produce peptides as a part of their defense mechanisms, signaling systems, or for various physiological functions. In recent years, scientists have been studying these peptides for their potential therapeutic benefits, including their ability to alleviate pain.

Animal peptides exhibit antinociceptive properties by interacting with the body’s pain-signaling pathways. They can modulate pain perception through various mechanisms, including interaction with receptors involved in pain transmission, inhibition of inflammatory processes, and modulation of neuronal excitability [39]. Understanding the specific mechanisms through which animal peptides exert their antinociceptive effects is crucial for developing targeted and effective pain management strategies.

One of the remarkable aspects of animal peptides is the diversity in their sources and structures. Venomous creatures, such as snakes, spiders [40,41], and cone snails, are well-known for producing peptides with potent bioactivities, including antinociceptive effects [42]. Additionally, peptides derived from insects or the skin secretions of amphibians have also shown promise in pain modulation.

### 2.1. Peptides Derived from Sea Snails

Among the venomous animals, snails are an important source of peptides with different biological activities. Several peptides with antinociceptive activity are characterized by the venoms of various snails [43,44,45]. The very interesting molecules with antinociceptive activity are conotoxins (Table 3). These peptides from cone snails are potent and highly selective blockers or modulators of ion channel functions [46].

For example, α-conotoxin RgIA, the peptide containing 13 amino acid residues with two intramolecular disulfide bonds (Table 3), was isolated from *Conus regius* [46]. It has been reported that in rat models of neuropathy, RgIA is a potent pain-relieving compound. Its effects are associated with modulating N-type Ca_v_2.2 VGCCs. Furthermore, the structure–activity relationship (SAR) studies led to the development of a new analog of RgIA (RgIA4—H-Gly-Cys-Cys-Thr-Asp-Pro-Arg-Cys-Cit-Tyr(3-I)-Gln-Cys-Tyr-OH) with high potency in humans and rodents [46].

Liu et al., described the strong antinociceptive activity of α-conopeptide, Eu1.6 from *Conus eburneus*. Eu1.6 exhibited antinociceptive activity in rat partial sciatic nerve injury and chronic constriction injury models, and its activity was more potent than that of a combination of morphine and gabapentin [47].

Other important classes of conotoxin peptides are the ω-conotoxins that block calcium channels [43]. The ω-conotoxin GVIA (27 amino acids peptide with three disulfide bonds) is the first peptide isolated from *C. geographus* [43]. The ω-conotoxins have also been isolated from many other species, such as *C. magus* and *C. catus*, forming a family of peptides 24–30 residues long and containing three intramolecular disulfide bonds (Table 3). The ω-conotoxin GVIA is a selective blocker of N-type voltage gated Ca_v_ (VGCCs) channels.

The most extensively analyzed ω-conotoxin is ω-conotoxin MVIIA (Table 3) from *C. magus* [43]. This peptide specifically blocks N-type Ca_v_ channels (Ca_v_2.2) and inhibits K^+^-induced [^3^H]-γ-aminobutyric acid (GABA) release in the hippocampus in vivo. This conotoxin has been approved by the Food and Drug Administration (FDA) as a non-opioid analgesic peptide against long-term neuropathic pain in humans under the commercial name of ziconotide or Prialt^®^ [48]. However, the ω-conotoxin GVIA is more potent than ω-conotoxins MVIIA or morphine. Several studies have reported that ziconotide does not interact with opioid receptors, and it does not cause analgesic tolerance or other opioid-induced systemic effects. However, its injection causes different CNS adverse effects, such as dizziness or memory impairment [48].

### 2.2. Peptides Derived from Spiders

Spider venom contains a broad range of peptide toxins, exerting several activities in biological systems. To date, a large number of peptides with antinociceptive properties have been isolated from several spider venoms [40,41,52]. Typical spider peptides are rich in disulfide brides, which form the inhibitor cystine knot (ICK) motif. Most contain six cysteine residues that form three disulfide bonds through connections between Cys^1^–Cys^4^, Cys^2^–Cys^5^ and Cys^3^–Cys^6^. However, spider peptides may also contain four or even five disulfide bonds [52]. More peptides have shown antinociceptive effects by modulating ion channels such as acid-sensing ion channels (ASICs), stretch-activated channels (SACs), or voltage-gated ion channels, including calcium (Ca_v_) and sodium (Na_v_) channels. Spider peptides may also activate receptors such as purinergic receptors (P2X3). Some of these neurotoxins could be the starting point for developing future analgesics.

Among antinociceptive toxins present in spider venoms are purotoxins. Purotoxin-1 (PT1) was isolated from the crude venom of the Central Asian Spider *Geolycosa* sp. [53,54]. This single-chain peptide contains 35 amino acid residues, including 8 cysteines involved in 4 intramolecular disulfide bridges (Table 4). Three disulfide bridges (Cys^3^–Cys^16^, Cys^10^–Cys^21^, and Cys^15^–Cys^32^) form the cystine knot, although there is also a loop secured by the disulfide bridge Cys^23^–Cys^30^. PT1 selectively inhibits P2X3 receptors, which are known to be implicated in pain mechanisms. Moreover, in the behavioral experiments carried out in rats, the antinociceptive effect was observed after injection of PT1 [53]. Purotoxin-2 (PT2), consisting of 64 amino acid residues with 4 disulfide bonds (Table 4) [54], was isolated from the venom of the same spider, and similar to PT1, it modulates P2X3 receptors.

The venom of the Brazilian armed spider *Phoneutria nigriventer* contains several potent peptide toxins. The most toxic components derived from this venom are neurotoxins PnTx2-6 (δ-CNTX-Pn2a) and PnTx2-5. These molecules contain 48 amino acid residues (Table 4) [55,71,72]. PnTx2-6 modulates voltage-gated Na^+^ channels and stimulates the production of nitric oxide (NO). However, PnTx2-6 induces nociception but is highly toxic; therefore, its therapeutic use is not possible. To overcome these limitations, the synthetic 19 amino acid peptide PnPP-19 was developed (Ac-GERRQYFWIAWYKLANSKKa) [72]. This molecule does not act on the Na_v_ channels but shows great potential as a therapeutic drug without significant toxicity. The PnPP-19 can act on the activation of DOR, MOR, or CB1 cannabinoid receptors. Moreover, the antinociception induced by this peptide appears to involve the inhibition of neprilysin, a neutral endopeptidase that prevents the destruction of endogenous opioids [72].

The next molecule isolated from the venom of the Brazilian armed spider, *Phoneutria nigriventer* [58,73], is Phα1β, also known as PnTx3-6. This neurotoxin is a small protein containing 55 amino acids and 6 disulfide bridges (Table 4). It was originally identified as an antagonist of two ion channels involved in nociception: the N-type voltage-gated calcium channel (Ca_v_2.2) and transient receptor potential ion channel (TRPA1). In animal models, the administration of Phα1β has been shown to reduce both acute and chronic pain. PnTx3-6 also produces a strong antinociceptive effect on cancer-related pain and causes minimal side effects at high doses. This peptide is more effective and potent as an antinociceptive agent than ω-conotoxins (peptides isolated from cone snail venom that inhibit Na_v_ channels with great potency and selectivity).

Brazilian armed spider venom also contains other peptides such as PnTx3-1, PnTx3-2, PnTx3-3, PnTx3-4, and PnTx3-5 (Table 4) [56]. These peptides inhibit voltage-activated calcium channels and induce antinociceptive effects. Recently, it was shown that PnTx3-1 also causes an antinociception by interfering with the activation of the cholinergic system [56]. The peptide PnTx3-3 is a 34-amino acid polypeptide (Table 4) [56,57]. It inhibits all known high-voltage activated calcium channels (L-, P/Q- and R-type currents) (Ca_v_), and most effectively, the P/Q- (Ca_v_2.1/CACNA1A) and R-type (Ca_v_2.3/CACNA1E) currents, and produces antinociception in models of neuropathic pain and inflammation. The sequence of PnTx3-5 consists of 36 amino acids (Table 4) [59]. It is a blocker of L-type VGCCs [59]. It was reported that the intrathecal administration of the peptide PnTx3-5 produced antinociception in postoperative, neuropathic, and cancer-related pain models. Moreover, antinociceptive effects induced by this peptide were observed at a dose much lower than morphine [59].

Another peptide toxin-peptide PnTx4(6-1) (Table 4), called δ-Ctenitoxin-Pn1a (δ-CNTX-Pn1a), was also isolated from that venom. It is a 48-amino-acid polypeptide with 5 disulfide bridges [56,60]. In various experimental models of pain, this peptide induced a clear antinociceptive effect in inflammatory, neuropathic, and nociceptive conditions. According to Emerich et al., in the nociceptive pain model, this effect seems to involve CB1 cannabinoid receptors as well as MOR and DOR, although the specific mechanisms have not been clarified [74]. Based on the amino acid sequence of PnTx4(6-1), a 13 amino acid analog PnAn13 (CDSYWSKSSKCREa) was synthesized, with a clear antinociceptive effect in rats, which involves the cannabinoid and opioid systems [75].

The β-theraphotoxin-Cd1a (β-TRTX-Cd1a) was isolated from the African rear-horned baboon tarantula *Ceratogyrus darling*, and it is a 33-amino acid peptide (Table 4) [61] that blocks Ca_v_2.2, Na_v_1.1–1.2, and Na_v_1.7–1.8 channels, with a higher potency for Na_v_1.7. Due to its ability to regulate ion channels and its antinociceptive effects, β-TRTX-Cd1a has potential therapeutic applications for developing peripheral pain treatment drugs [61].

The venom of the Chinese tarantula *Chilobrachys guangxiensis* contains a family of peptides named Jingzhaotoxins (JzTx). The JzTx-34 is a 35-residue polypeptide (Table 4) that inhibits Na_v_1.7 channels, exhibiting antinociceptive properties with higher affinity as compared to all other subtypes of Na_v_ [62]. This toxin also inhibits voltage-gated potassium channels (K_v_) in rat dorsal root ganglion (DRG) neurons. Moreover, JzTx-34 shows a longer duration, and it is more effective than morphine in rodent pain models [76].

Hainantoxin-IV, also termed μ-TRTX-Hhn1b (HNTX-IV), is a 35-residue peptide (Table 4) from the Chinese bird spider *Ornithoctonus hainana* (*Haplopelma hainanum*) venom [63]. The native μ-TRTX-Hhn1b inhibits Na_v_1.2, Na_v_1.3, and Na_v_1.7. The antinociceptive effect of μ-TRTX-Hhn1b is attributed to its ability to inhibit the tetrodotoxin-sensitive (TTX-S) voltage-gated sodium channels (VGSCs), especially human Na_v_1.7 expressed in HEK 293 cells. It was reported that the activity of this peptide in rat models of inflammatory and neuropathic pain might be comparable or superior to morphine and mexiletine [63].

Several peptide toxins were purified from the venom of the tarantula *Ornithoctonus huwena* and named huwentoxins [64,65,66,77]. Huwentoxin-XVI (HWTX-XVI) contains 39 amino acid residues and 3 disulfide bridges (Table 4) [66]. This molecule is a specific blocker of N-type VGCCs in DRG rat neurons. HWTX-XVI induces a slight but significant antinociceptive effect on thermal pain in rat formalin tests. Moreover, after injection of this peptide, pain reduction occurred immediately and lasted longer, while morphine-induced pain reduction was immediate but lasted for a shorter time.

SNX-482 peptide (Table 4) was isolated from the venom of the African tarantula *Hysterocrates gigas* [67]. As the R-type channel selective blocker, it causes antinociception in neuropathic pain models. Moreover, this peptide inhibits the N-type Ca_v_2.3 at the nanomolar range and the L-type Ca_v_1.2 channel at the low micromolar range. Some results indicate that SNX-482 inhibits neuronal responses in a neuropathic pain model; therefore, it is possible that SNX-482 can be used to reduce dorsal horn neuronal pain in neuropathic pain therapy.

Another interesting spider peptide is phlotoxin 1 (Ph1Tx1), a promising antinociceptive peptide with a high affinity for Na_v._ It is a 34-residue toxin purified from *Phlogiellus spider* venom (Table 4) [68]. Phlotoxin-1 is a potent blocker of the Na_v_1.7 channel, which was identified as the most sensitive ion channel for Ph1Tx1.

It has already been demonstrated that agatoxins (μ-Aga-I and ω-agatoxin IVA) from the venom of the funnel web spider *Agelenopsis aperta* possess an antinociceptive effect. The ω-agatoxin IVA is 48 amino acid polypeptide (Table 4) [69], and it is a specific blocker of the voltage-gated calcium channels (Ca_v_2.1) in vertebrate central neurons.

Liang et al., isolated from the hard tick’s synganglia (central nervous system), *Amblyomma testindiarium*, an opioid peptide, which shares similarities with mammalian hemorphins (Table 4). This peptide displayed a dose-related antinociceptive effect in mice [70].

### 2.3. Peptides from Scorpion Venom

Numerous antinociceptive peptides have been isolated from scorpion venoms. These peptides exhibit various biological and pharmacological activities [78]. The first peptide from the venom of the Chinese Scorpion *Buthus martensi* Karsch (BmK) with an antinociceptive activity was described in 1994, and now more than 10 peptides with antinociceptive effect in mice and rats have been identified: BmK I1, BmK I4, BmK I6, BmK AngP1, BmK dITAP3, BmK ANEP, BmK Ang P1, BmK AS, BmK AS-1, BmK AGP-SYPU1, BmK AGP-SYPU2, BmK AGAP, etc.

BmK AGAP is a long-chain peptide with 66 amino acid residues (Table 5). It was observed that BmK AGAP has antitumor activity and a weak inhibitory effect on the voltage-gated sodium channel Na_v_1.7. Therefore, this channel may not be the primary target of BmK AGAP. Moreover, the study also suggests that BmK AGAP inhibits the transient receptor potential vanilloid-1 (TRPV1) and the potassium channels KCNQ2/3 currents, and intrathecal injection of this venom peptide with lidocaine produces long-lasting antinociception [79].

The high homology to BmK AGAP shows BmK AGP-SYPU2 (Table 5), differing in only four amino acids: Lys^2^, Val^7^, Glu^27^, and Arg^62^ [80]. BmK AGP-SYPU2 exhibits strong antinociceptive effects against both visceral and somatic pain and shows antitumor activity in mice. However, it has stronger antitumor activity and weaker antinociceptive activity compared with those of BmK AGAP. Similarly, highly homologous peptides BmK AS and BmK AS-1, containing 66 amino acid residues (Table 5), induce strong central and peripheral antinociceptive effects in rats. Peptide BmK AS modulates voltage-gated Na^+^ channels of animals [78]. It is interesting that the amino acid sequence of BmK AS-1 indicates a structural homology to AaH IT4 isolated from the venom of African Scorpion *Androctonus australis* Hector.

Peptide BmK IT2, derived from venom of *Buthus martensi* Karsch, is composed of 61 amino acids with 4 disulfide bonds (Table 5) [78,82]. Peptide BmK-YA contains an enkephalin-like sequence and activates three subtypes of opioid receptors, MOR, DOR, and KOR, but with selectivity for the DOR subtype [83].

TsNTxP was isolated from the venom of the scorpion *Tityus serrulatus.* It exhibits antinociceptive effects in rodents by inhibiting glutamate release and stimulating the voltage-gated sodium channels. It is structurally similar to neurotoxins that interact with Na_v_ channels, although it is non-toxic [78].

The venom of scorpion *Heterometrus laoticus* contains peptide hetlaxin, showing both antinociceptive and antiinflammatory activity (Table 5). Hetlaxin possesses a high affinity to the K_v_1.3 potassium channel [86].

### 2.4. Peptides Isolated from Other Arthropods

In recent years, an increasing number of insect peptides have been proving useful in several pharmaceutical applications. Several insect peptides with different amino acid sequences and the length of the peptide chain were reported to display the antinociceptive effect, such as proctolin [87], leucopyrokinin [88], trypsin modulating oostatic factor (TMOF) [89], alloferon [90] and myotropins peptide [91]. This activity was at least in part mediated by the central opioid system as it was blocked by naloxone—an opioid antagonist. Moreover, the pentacosapeptide poneratoxin, discovered in the venom of ant *Paraponera clavata*, also exerts an antinociceptive effect in rats [92]. However, this effect does not seem to be mediated by the central opioid system but perhaps by central neuronal sodium channels, central nicotinic receptors, or NO• radicals.

The literature data indicate great potential for studying the neuroactive compounds isolated from Hymenoptera in the treatment of pain [93]. The antinociceptive compound [Thr^6^]-bradykinin (Thr^6^-BK) (Table 6) was isolated from the venom of wasp *Polybia occidentalis* [93]. It exerts a strong effect on i.c.v. administration in rats. Pallipin-III, isolated from the social wasp *Agelaia pallipes pallipes*, is a 22-amino acid peptide that exhibits antinociceptive and anti-inflammatory effects when injected peripherally into mice (Table 6).

Galante et al., identified a 12-amino acid long peptide (Table 6) called protonectin from the venom of the wasp *Parachartergus fraternus* [95]. This peptide induces the antinociceptive effect, but it may cause some sort of toxicity. However, its analog, protonectin-F, containing the phenylalanine residues in positions 2 and 8, has increased bioavailability and antinociceptive effect comparable to that of morphine [95]. Gonçalves et al., identified the peptide Agelaia-MP I from the same wasp [94]. It is interesting that the sequence is identical to the mastoparan peptide described in another wasp species, *Parachartergus fraternus*. Agelaia-MP I displayed dose-dependent antinociceptive activity when injected directly into the CNS of mice. The biological results suggest that this peptide may act on non-opioid receptors.

Bee venom from *Apis mellifera* is a complex mixture that includes proteins and peptides such as melittin or adolapin [97]. Adolapin is a polypeptide with 103 amino acid residues, known for its antinociceptive properties [98]. Melittin evokes antinociceptive effect in chemotherapy-induced peripheral neuropathy. This effect is mediated by activating the spinal α1 and α2-adrenergic receptors [99].

### 2.5. Peptides of Amphibian Origin

It has been demonstrated that some bioactive peptides from amphibians, such as bradykinins, tachykinins, and cholecystokinins, exert algesic effects [100,101]. Dermorphins and deltorphins (Table 7) are the family of antinociceptive peptides that have been found in the skin of South American frogs [25]. Dermorphins are the most potent and selective MOR agonists, whereas deltorphins are peptides with high affinity and selectivity for DOR [102,103]. The antinociceptive peptide was also extracted from the brain of the frog *Odorrana graham*. So far, the molecular mechanism of the antinociceptive effect of odorranaopin is unknown [104].

### 2.6. Peptides from Snake Venom

Several studies reported that peptide toxins purified from snake venom have the potential to become new painkillers [39].

Three isoforms of mambalgin peptides have been isolated from the venom of black (*Dendroaspis polylepis*) and green mamba (*Dendroaspis angusticeps*) (Table 8) [108]. Mambalgin-1,-2,-3 evoke antinociceptive effects after local subcutaneous injection or after injection into the central nervous system. These peptides block a set of acid-sensing ion channels to relieve pain.

It has been reported that crotalphine a 14-amino acids peptide with a pyroglutamic acid and a disulfide bond (Table 8), isolated from the venom of the South American rattlesnake *Crotalus durissus terrificus*, produces an antinociception by displaying opioid activity [110]. It is worth noting that the antinociceptive effect of crotalphine is blocked by the KOR antagonist nor-binaltorphimine and partially reversed by *N*,*N*-diallyl-Tyr-Aib-Phe-Leu-OH, DOR antagonist, whereas in a PGE2-induced hyperalgesia model the activity is reversed by intraplantar injection of dynorphin A antiserum, indicating the involvement of endogenous opioids [112,113]. It is interesting that crotalphine has a higher antinociceptive activity compared to morphine.

The μ-EPTX-Na1a is a 62-residue polypeptide (Table 8) from the venom of the Chinese cobra (*Naja atra*). It was shown to be a potent inhibitor of the voltage-gated sodium channel Na_v_1.8 subtypes and hence contributes to reducing inflammatory and neuropathic pain [111]. In vivo, in rodent inflammatory and neuropathic pain models, μ-EPTX-Na1a attenuates nociceptive behavior more strongly than morphine.

The α-cobratoxin (α-CbTX) (Table 8) has been isolated from the venom of the cobra snake *Naja kaouthia*. It was suggested that the analgesic effect α-CbTX, independent of the opioid system, occurs by blocking T-type VGCCs [48,109].

The interactions between receptors and ligands open the possibilities of intervention based on the design of agonists and antagonists modifying the selectivity and intensity of response [114]. Peptides, being natural ligands, serve as templates in search for novel biomodulators. GPCR-targeted peptide-based drug discovery aimed at antinociception uses peptides of animal origin to develop new structures [35]. The opportunities resulting from studies of venoms and toxins, supported by genomics, peptidomics, and other omics, deliver structural motives to be used in the molecular modeling of potential drug candidates. The diversity of peptides and structural opportunities are combined with the flexibility of peptide synthesis based on solid-support strategies or in-solution transformations. Modular peptide assembly delivers the desired compounds, frequently containing non-proteinaceous amino acids or post-translational modifications. Larger structures could be formed by conjugation or biotechnological procedures [115]. Other advantages of peptides result from the compatibility with regular metabolism and lack of accumulation. Synthetic opportunities and the predictability of metabolic fate are the main advantages of peptides when compared with small molecules in drug development [35].

The pharmacological disadvantages of peptides, including limited stability, oral bioavailability and biodistribution, could be overcome by modification strategies presented in the following parts of the text.

## 3. Structural Modifications of Opioid Peptides

In the last two decades, the interest in opioid peptides as potential drug candidates has increased significantly. Peptides generally are characterized by relatively high activity, selectivity, and low toxicity. However, the native opioid peptides have several limitations, such as low metabolic stability, poor bioavailability, and low blood–brain barrier (BBB) permeability, which discriminate them as therapeutic agents for clinical use [31,116,117]. Most peptides cannot be administered orally as gastrointestinal enzymes rapidly inactivate them; therefore, subcutaneous or intravenous administration is required. The very low brain permeability or inability to cross BBB limits the access of exogenously administered peptides to the required site of action in the brain.

To overcome these problems and to optimize the therapeutic potential, various strategies have been proposed to develop novel analogs of opioid peptides with a better pharmacological profile than the native compounds, i.e., increased metabolic stability, bioavailability and/or selectivity of the site of action (central or peripheral action) [3,11,31,32,33,116,117,118]. The main chemical modifications in opioid peptidomimetic design include incorporating unnatural or d-amino acids, forming non-peptide bonds, N/C-terminal modifications, cyclizing linear peptides, and synthesizing conjugates and hybrid structures. These methods have proven to be effective in achieving high selectivity and specificity [31,32,33,116,117,118,119,120].

Extensive structure–activity relationship (SAR) studies of opioid peptides are focused on the development of centrally acting analogs that cross the blood–brain barrier, which would produce an analgesic effect as strong as morphine without causing addiction or tolerance. In this case, particular emphasis is placed on obtaining lipophilic peptides that will penetrate the BBB and activate MOR in CNS. On the other hand, compounds that do not cross BBB are also interesting, especially when a local antinociceptive effect is required, e.g., in the inflammation processes in the gastrointestinal tract [121,122].

The three families of endogenous opioid peptides, β-endorphins, dynorphins, and enkephalins (ENKs), share a similar amino acid sequence of Tyr-Gly-Gly-Phe-X. Due to their structural homology and natural flexibility, endogenous linear opioid peptides may interact with multiple receptor subtypes.

**Figure 1:**
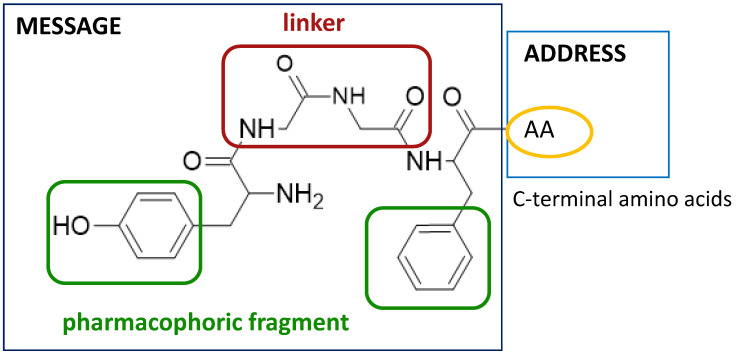
Message and address regions of natural opioid peptides.

Natural opioid peptides contain aromatic amino acids that are generally recognized as key pharmacophoric residues. According to the message–address concept [123], the *N*-terminal tri- or tetrapeptide is responsible for the biological activity, whereas the address region is the variable structure responsible for selectivity. The address region is the variable structure responsible for selectivity (Figure 1). SAR research has shown that the interactions with opioid receptors have strict requirements for the message sequences, whereas the stability of the peptides is more dependent on the address sequence. The key pharmacophoric residues in the message region of opioid peptides are Tyr and Phe, and their Nα-amino, phenolic, and aromatic groups are critical for activity. Therefore, the tyrosine residue is not typically involved in structural modifications of opioid peptides [31].

### 3.1. The First Step: Amino Acid Replacement

For decades, researchers have been developing opioid peptide analogs as drug candidates. Studies on SAR of opioid peptides in the 1970s showed that substituting D-Ala at position 2 in enkephalin analogs increased their stability and activity [124]. In addition, the incorporation of D-Ala, MePhe, and Met(O)-ol at positions 2, 4, and 5 of [Met]enkephalin, respectively, results in a remarkable increase in antinociceptive efficacy. A comparable result can be achieved by replacing the peptidase-susceptible peptide bond with a dipeptide isostere such as an olefin, ester, or triazole (Table 9). According to a recent study, the use of β-Ala at the *N*-terminal amide of an ENK-like tetrapeptide results in longer-lasting antinociceptive effects due to reduced availability of endopeptidases [125]. Another study demonstrated that the addition of an *N*-terminal *N*α-guanidyl group to form guanidyl-Tyr-D-Ala-Gly-Phe-Leu-tetrazole, in combination with a *C*-terminal tetrazole and D-Ala, significantly enhances stability, lipophilicity, affinity, and potency [126]. SAR studies allow the identification of changes in the amino acid sequence that will result in a viable drug candidate. Amino acid residues included in pharmacophores can be replaced with a residue that modifies the receptor binding and biological activity of the peptide, whereas residues that are enzymatically unstable but do not significantly affect the overall biological activity of the peptide can be replaced with more stable moieties. Applying these principles, new linear analogs, DADLE and DSLET (Table 9), have been developed. These examples of enkephalin structural analogs exhibit higher selectivity and agonist activity for DOR. Research has shown that agonist activation of DOR does not produce as potent an antinociceptive effect as MOR but is likely to cause fewer side effects. Another example is DAMGO, a synthetic derivative of [Met]enkephalin. Although native [Met]enkephalin activates both receptors non-selectively, DAMGO is a highly selective MOR agonist [127].

Dermorphin and enkephalin analogs containing *N*-terminal derivative of tyrosine with two methyl groups at the 2′,6′ positions of the aromatic ring but without amino group (deaminated Dmt) were found to be moderately potent antagonists of DOR and MOR. It can be concluded that the amino group is not necessary for receptor binding, and its removal in agonistic opioid peptides containing the regular *N*-terminal 2′,6′-dimethyltyrosine (Dmt) residue may be a general method to convert them into antagonists [136]. The dermorphin-derived peptide H-Dmt-d-Arg-Phe-Lys-NH_2_ ([Dmt^1^]DALDA) (Table 9) [133] is a highly potent MOR agonist with subnanomolar MOR binding affinity. It is systemically active (crosses the blood–brain barrier) and, in comparison with morphine, it has much higher antinociceptive potency in both acute and neuropathic pain models [137,138].

Endomorphins differ from other endogenous opioids in their sequence. The opioid receptor motif in endomorphin-1 (EM-1) differs from the typical Tyr-Gly-Gly-Phe-X pattern, as it has the sequence H-Tyr-Pro-Trp-Phe-NH_2_. Similarly, endomorphin-2 (EM-2) has the sequence H-Tyr-Pro-Phe-Phe-NH_2_. The presence of Pro in position 2 induces a turn in the peptide structure, allowing the aromatic groups Tyr and Phe/Trp to bind to the MOR receptor. Modifications to increase enzymatic stability have primarily involved the replacement of the Pro residue. The introduction of d-Pro significantly decreased the binding affinity to the receptor, but the addition of unnatural amino acids with six-membered heterocyclic rings, such as piperidine-2, 3-, and 4-carboxylic acids, which mimic Pro, significantly increased the metabolic stability and antinociceptive activity of the analogs [31].

The main limitation that must be overcome in designing centrally active peptides is their poor ability to penetrate the blood–brain barrier [139]. One way to improve the permeability of the BBB is to increase the lipophilicity of the therapeutic agent by removing polar groups from the side chains of the amino acids. However, studies have shown that the removal of the Tyr hydroxyl group in endomorphin results in a complete loss of activity. Another method is to add hydrophobic groups, such as methyl or acetyl, to increase the lipophilicity of the peptide. *N*-methylation effects of subsequent amino acid residues in the EM-2 molecule and the introduction of methyl groups to the aromatic rings of Tyr and Phe have been studied over the years. The 2′,6′-dimethyltyrosine (Dmt) analog, obtained by methylation of the phenolic tyrosine ring, resulted in a significantly increased level of bioactivity. The lipophilicity, permeability of BBB, and activity can also be increased by guanidino-addition and chlorohalogenation of amino acid side chains [140].

Currently, peptides that have mixed opioid receptor profiles represent an interesting new generation of candidates for peptide-based analgesics with improved potency and/or reduced side effects [141,142,143]. The first peptidic MOR opioid agonist/DOR antagonist H-Dmt-TicΨ[CH_2_NH]Phe-Phe-NH_2_ (DIPP-NH_2_[Ψ]) was reported by Schiller and co-workers. DIPP-NH_2_[Ψ] produced a potent antinociceptive effect, no physical dependence, and less tolerance than morphine [144]. In the following decades, extensive structure–activity relationship studies of opioid receptor ligands concentrated on obtaining analogs with high selectivity for one opioid receptor type. Indeed, the strongest antinociceptive effects, but also undesired side effects, are mostly mediated through the activation of MOR [2,11]. However, DOR and KOR can also contribute to the analgesic effects of opioids [142,145]. Now, it is widely accepted that simultaneous activation of multiple opioid receptors may result in additional analgesia with fewer side effects [143,145].

KOR selective agonists produce analgesia accompanied by some dysphoric effects [146], and this property limits their therapeutic development. However, the MOR/KOR agonists of the alkaloid structure, such as ethylketazocine (EKC), are safer than selectively acting agonists and have been used to treat cocaine addiction [147].

DOR agonists have been found to enhance the antinociceptive effects of MOR agonists, while DOR antagonists inhibit the development of tolerance and addiction, side effects of MOR agonists [145]. This observation resulted in the search for ligands that act simultaneously on two receptors, such as MOR agonist/DOR agonist or MOR agonist/DOR antagonist [148].

### 3.2. Biphalin—A Prominent Example of SAR Study

**Figure 2:**
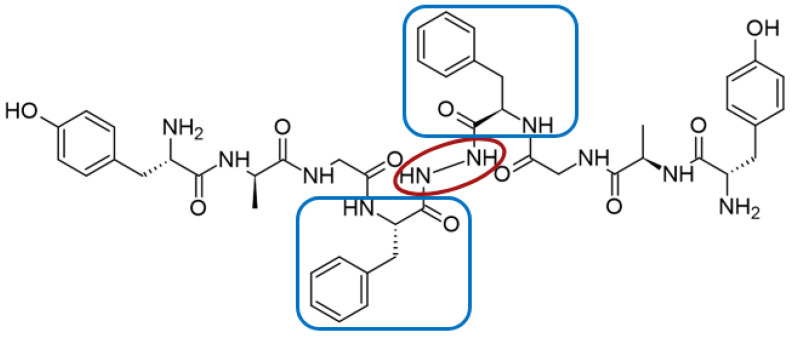
Structure of biphalin. The red mark indicates the hydrazide bridge. The blue mark indicates the phenylalanine.

Biphalin, (H-Tyr-d-Ala-Gly-Phe)_2_hydrazine, a dimeric analog of two enkephalin-like fragments connected “tail-to-tail” via a hydrazine bridge, is the best-known and the most studied example of a peptide ligand with the dual MOR/DOR agonist profile [149] (Figure 2). Biphalin displays exceptionally high antinociceptive activity, induces analgesia in acute, neuropathic, and chronic animal pain models, and is 1000 times more potent than morphine when administered intrathecally. Moreover, in tests conducted on rodents after long-term administration, biphalin induces less physical dependence and withdrawal syndromes than morphine [150,151]. Despite its excellent antinociceptive activity, the clinical use of this compound is limited due to its poor penetration of the BBB and/or low metabolic stability. To overcome these limitations and to obtain compounds with modified BBB penetration and better pharmacological profile, several analogs based on the structure of biphalin have been designed and synthesized. The modifications include the reduction of the length of the peptide chain in one biphalin arm, modification of the hydrazide bridge, replacement of amino acid residues in positions 4,4′ and 3,3′, conjugation with polyethylene glycol (PEG), the introduction of fluorescent residues, and cyclization [152,153].

Different fragments of biphalin and their analogs were modified by the replacement of Phe^4^ by Trp, whereas the Phe residue in the second arm was replaced by d-Phe, l/d-Nle (norleucine), Tyr, or Trp. SAR analysis revealed that H-Tyr-d-Ala-Gly-Phe-NH-NH<-Phe is the minimal fragment necessary to express equal affinities and the same biological activity profile as the parent biphalin. A shorter fragment, H-Tyr-d-Ala-Gly-Phe-NH-NH_2_ exhibits good affinity for MOR, similar to biphalin, but the affinity for DOR is 100 times lower. Moreover, it was shown that the presence of the *N*-terminal Tyr is crucial for biphalin’s high affinity with opioid receptors and its pharmacological activity [153].

In order to determine the influence of hydrazine linkers on biphalin opioid receptor affinity and its activity, several biphalin analogs with different non-hydrazine linkers were obtained [149,154,155]. Modification of the hydrazide bridge by using alkyl diamines of variable length (CH_2_)_n_ led to reduced activity, probably because of the higher degree of freedom around the diamide bridge positioning the two pharmacophores in the receptor pockets [149,154].

**Figure 3:**
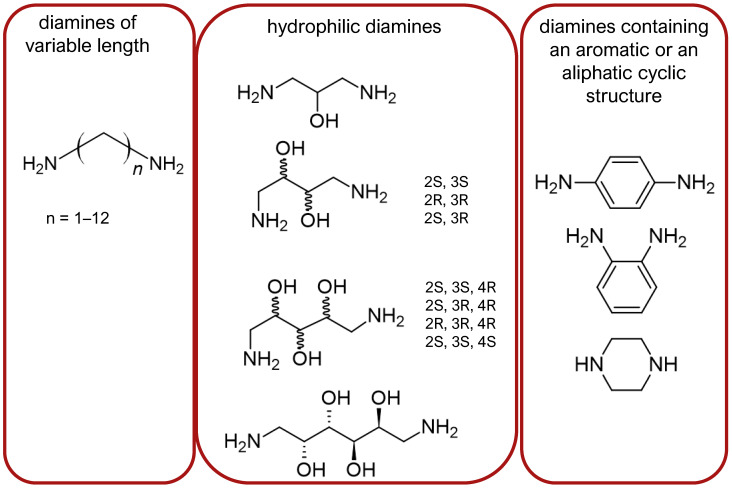
Selected examples of bridges connecting peptide fragments in new biphalin analogs.

Stepinski et al., used diaminepolyoles for connecting biphalin motives (Figure 3) and demonstrated that the use of hydrophilic spacers creates new possibilities in the modulation of activity and selectivity of opioid peptide bivalent ligands, and both the length and configuration of these spacers are important factors in determining receptor potency and selectivity, but still accompanied by a loss of activity [156].

Mollica and co-workers [155] synthesized biphalin analogs, in which the hydrazide bridge was replaced with diamines containing an aromatic or an aliphatic cyclic structure: 1,4-phenylenediamine, 1,2-phenylenediamine, and piperazine. These new analogs showed better affinity and in vitro bioactivity than biphalin itself, thus suggesting that the high activity of biphalin is not critically related to the structural and conformational properties of the hydrazide bridge and that hydrazine bridge can be conveniently substituted by different conformationally constrained cycloaliphatic and cycloaromatic diamine linkers (Figure 3).

To enhance the message-address interactions between the ligand and the receptors and thus increase the activity of biphalin, the modifications of amino acid residues were made in the peptide chain. It has been demonstrated that substituting Phe residue in position 4 of enkephalins can significantly affect binding to the MOR and DOR. Misicka et al., synthesized a series of biphalin analogs in which different substituents in the para position of the aromatic ring of Phe residue (-NO_2_, -Cl, -F, -I, and -NH_2_) were introduced [157]. Symmetrical incorporation of pF or pNO_2_-Phe residues in position 4 of biphalin (see Figure 2) resulted in analogs that showed, although to a different degree, enhancement of affinity towards DOR and MOR accompanied by an increase of DOR/MOR selectivity [157,158]. Different non-hydrazine linkers were incorporated with the pF-Phe to improve BBB permeability [159] further. The introduction of piperazine linker and pF-Phe led to the analog with remarkable in vitro and in vivo activity. A new compound (H-Tyr-d-Ala-Gly-(pF)Phe)_2_-piperazine, named AM94, showed high binding affinity at MOR and DOR and a greater and longer-lasting antinociceptive effect than biphalin after different administrations routes such as intrathecal and subcutaneous and was one of the most potent linear biphalin analog described.

Substituting α-amino acids with their β-isomers in biologically active peptides may result in increased enzymatic stability and a strong influence on peptide conformation. The replacement of Phe by β3 homoamino acid (hβ3-Phe) resulted in a biphalin derivative with good MOR- and DOR affinities and antinociceptive activity in vivo together with increased enzymatic stability in human plasma [160]. Incorporation of hβ3-pNO_2_-Phe and a 1,2-phenylenediamine or hydrazine bridge also leads to active and selective analogs, with a selectivity depending on the type of linker. The compound with a hydrazine linker showed noticeable binding selectivity to MOR, while the peptide with a 1,2-phenylenediamine linker had slight DOR selectivity. Both analogs produced a greater antinociceptive effect compared to morphine after i.t. administration [161].

### 3.3. Cyclic Analogs: Frozen Structure

Among various synthetic approaches, cyclization is a powerful tool in peptide chemistry that is well-recognized and used to develop peptidomimetics with improved pharmacological properties. Cyclic analogs adopt more strict conformations that are better defined than those of their linear counterparts, which often results in higher receptor binding affinity, metabolic stability, and increased lipophilicity, usually followed by improved BBB permeability [119,120]. Linear sequences can be cyclized using different strategies and in different positions by forming bonds between peptide ends or side chains. Apart from disulfide bridges in peptides containing cysteine residues, the most often used bridging methods between side chains are amide bonds and urea bonds [119]. The first cyclic analogs of opioid peptides (enkephalin analogs) were synthesized already in the 1980s [162,163,164]. In the last 50 years, a great number of cyclic opioid peptides have been synthesized in the hope of designing a new generation of peptide-based analgesics [119,120].

MOR selective endomorphins and morphiceptin analogs are difficult to cyclize due to their short tetrapeptide sequence and the lack of reactive side-chain groups. Therefore, different approaches have been used to obtain cyclic analogs of such peptides, such as extending a peptide chain by introducing additional amino acids or/and functionalizing amino acid side chains.

In the search for new MOR-selective opioid peptide analogs with improved analgesic profiles, Janecka and co-workers described several series of endomorphins and morphiceptin analogs of a general formula H-Tyr-c[Xaa-Phe-Phe-Yaa]-NH_2_ or H-Tyr-c[Xaa-Phe-d-Pro-Yaa]-NH_2_, respectively (where Xaa, Yaa = l/d-Asp or l/d-Lys), obtained by cyclization through an amide bond between the side-chain of bifunctional amino acids Lys and Asp introduced into the peptide chain in positions 2 and 5, respectively [165,166,167]. New cyclic peptides were designed to preserve the most important structural elements necessary for binding endomorphin-2 and morphiceptin with MOR, which are amino and phenolic groups of Tyr^1^ and the aromatic ring of Phe^3^ [168]. Some of those new analogs showed higher MOR affinity than endomorphin-2 or morphiceptin and produced a strong and long-lasting antinociceptive effect in hot plate test in mice after intracerebroventricular (i.c.v.) administration. Among others, analog H-Tyr-c[d-Lys-Phe-Phe-Asp]-NH_2_ displayed greatly improved stability in the rat brain homogenate and produced antinociception also after intravenous (i.v.) administration, which showed that it was able to cross, at least to some extent, the blood–brain barrier [166]. Cyclic pentapeptides mentioned above became parent compounds for further structural modifications aimed at obtaining analogs with increased metabolic stability and BBB permeability [121,167,169,170].

Perlikowska et al., modified the sequence of analog H-Tyr-c[d-Lys-Phe-Phe-Asp]-NH_2_ by the introduction of d-1- or 2-naphthylalanine residues (d-1-Nal and d-2-Nal, respectively) in position 3 [167]. Both analogs showed high MOR affinity and agonist activity and were enzymatically stable. Unfortunately, the increase in lipophilicity was achieved at the expense of water solubility. The analog H-Tyr-c[d-Lys-d-2-Nal-Phe-Asp]-NH_2_ showed a strong antinociceptive effect when given i.c.v., but could not be tested after i.v. administration where higher concentrations of the compound are required.

The lipophilic character of a peptide can be increased by introducing methyl groups into the aromatic rings of Tyr and Phe. The replacement of Tyr in position 1 by highly lipophilic unnatural amino acid 2′,6′-dimethyl-L-tyrosine (Dmt) resulted in analogs with increased affinity at MOR, drastically improved in vitro stability and very strong antinociceptive activity after i.c.v. injection [121,169]. However, the i.v. administration of these peptides did not produce any significant antinociceptive effects, indicating that they did not cross the BBB. More detailed studies of analog H-Dmt-c[d-Lys-Phe-Phe-Asp]-NH_2_ (C-36) using the parallel artificial membrane permeability assay (PAMPA), considered as an accurate passive diffusion permeability model for BBB confirmed its very low permeation through this artificial barrier, suggesting that enhanced lipophilicity is not the only factor deciding of the blood–brain barrier permeability [121,169]. As opposed to C-36, Tyr^1^-containing analog H-Tyr-c[d-Lys-Phe-Phe-Asp]-NH_2_ could reach the brain after i.v. administration. Recently, Zadina et al. [171] reported a series of cyclopeptides of a similar structure also containing Tyr^1^ that were able to produce strong antinociception after peripheral administration. Therefore, the presence of Dmt^1^ in cyclic peptides may be a structural element responsible for the lower permeability of such analogs through biological barriers.

Based on the structure of the analog C-36 H-Dmt-c[d-Lys-Phe-Phe-Asp]-NH_2_, Perlikowska and co-workers synthesized a series of analogs, in which Phe^3^ and Phe^4^ residues were consecutively replaced by 2′, 3′ or 4′-methylphenylalanine (MePhe) [170]. Analogs with MePhe in position 4 showed an order of magnitude increased MOR affinity as compared with a parent compound C-36 and were strong MOR/KOR agonists and weak DOR agonists. In the in vivo hot-plate test in mice, the MePhe^4^-modified peptides showed remarkable antinociceptive activity after i.c.v. administration, which was most likely due to the concomitant activation of more than one opioid receptor type.

The Phe residues in position 3 or 4 in C-36 were also modified by the introduction of d-1-Nal and d-2-Nal [172]. Cyclopeptides with d-1-Nal and d-2-Nal in position 4 retained the sub-nanomolar MOR and nanomolar KOR affinity, similar but not better than that of a parent cyclopeptide. Analogs H-Dmt-c[d-Lys-Phe-d-1-Nal/d-2-Nal-Asp]-NH_2_ displayed high antinociceptive activity in mice not only after i.c.v. but also after systemic intraperitoneal (i.p.) injection, indicating that they were able to cross the BBB.

Piekielna et al., introduced fluorinated amino acids: 4-fluorophenylalanine (pF-Phe), 2,4-difluorophenylalanine (2,4-F-Phe), or 4-trifluoromethylphenylalanine (pCF_3_-Phe) into the sequence H-Tyr/Dmt-c[d-Lys-Phe-Phe-Asp]-NH_2_ instead of the Phe residue in position 3 or 4 [173]. Depending on the fluorinated amino acid residue and its position in the sequence, analogs were mixed with high-affinity MOR/KOR agonists, MOR/DOR/KOR agonists, or selective KOR agonists. The most potent analogs containing mono- and difluorinated Phe residues were full MOR and partial KOR agonists, showing high potency at the MOR and KOR and much lower potency and efficacy at the DOR. In hot plate tests in mice, these compounds produced strong antinociceptive effects after i.c.v. and i.p. administration [173].

Incorporation of trifluoromethyl group into the aromatic ring of Phe in the sequence of C-36 led to peripherally restricted opioid analog H-Dmt-c[d-Lys-Phe-(pCF_3_)Phe-Asp]-NH_2_ (F-81). This compound exhibited dose-dependent antinociceptive activity, significantly stronger than that of endomorphin-2 after i.c.v. administration, when given directly to CNS. On the other hand, peripheral i.v. administration of the peptide produced only a negligible antinociceptive effect in the hot plate test in mice, indicating that it could not cross BBB. The very low BBB permeability was also confirmed in the PAMPA test. After i.p. administration, analog F-81 significantly attenuated inflammation in the mouse models of colitis and showed strong antinociceptive activity in the mouse model of abdominal pain [121]. Such peripherally restricted opioid analgesics can be useful as a safer alternative for treating inflammatory painful disorders of the gastrointestinal tract.

In a search of endomorphins analogs with a low risk of adverse side effects, Zadina and co-workers obtained novel cyclic analog H-Tyr-c-[d-Lys-Trp-Phe-Glu]-Gly-NH_2_ (ZH853). The new compound showed high affinity, selectivity, and potent activation of the MOR. It was metabolically stable, penetrated the BBB, and had a strong antinociceptive effect after various routes of administration in multiple pain models. Compared to morphine, ZH853 showed dramatically improved antinociception-to-side-effect ratios, including reduction of respiratory depression, abuse liability, tolerance, motor impairment, and inflammation. The authors suggested that ZH853 could provide effective therapy for a diverse spectrum of pain conditions as well as reduce the opioid overdose epidemic [171,174].

The presented modification strategies, especially cyclization, were applied to biphalin. In Section 3.2, various analogs of linear biphalin were discussed, whereas here, we would like to present recent studies focused on the cyclization of biphalin by the incorporation of an additional bridge between biphalin arms.

**Figure 4:**
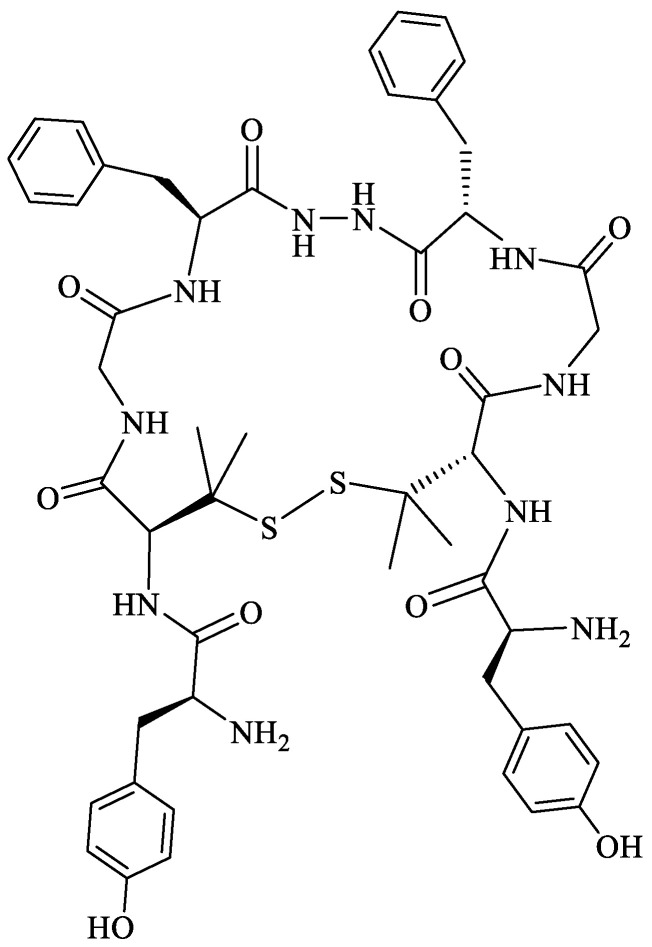
Structure of (H-Tyr-c[d-Pen-Gly-Phe])_2-_hydrazine.

To overcome the moderate stability of biphalin in human plasma, several cyclic analogs of biphalin with a disulfide linkage [152,175] or a xylene bridge were developed [176,177]. The first cyclic analogs of biphalin were synthesized by Mollica and co-workers in 2006. d-Ala residues in position 2,2′ of parent peptide were replaced by l/d-cysteine, and an intramolecular disulfide bond between the cysteine thiol groups was introduced [152], resulting in two analogs (H-Tyr-c[l/d-Cys-Gly-Phe])_2_-hydrazine (Figure 4). The introduction of a disulfide bridge was a step forward in revealing the active conformation at the binding pocket, leading to more potent compounds than the parent linear peptide [152,175]. In the next step, the native d-Ala residues were substituted with l/d-Pen (l or d-penicillamine, β,β-dimethylcysteine). Compound with l-Pen was scarcely inactive, whereas the analog (H-Tyr-c[d-Pen-Gly-Phe])_2_-hydrazine with d-Pen showed excellent MOR/DOR receptor affinity and a very good in vivo antinociceptive activity, its analgesic effect after i.c.v. injection was several times higher than for morphine.

After i.v. administration (“hot plate” and “tail-flick” tests), the new compound displayed a greater and longer-lasting antinociceptive effect than biphalin, thus suggesting a likely improvement of the pharmacokinetic parameters as compared to biphalin. However, its antinociceptive activity after i.v. injection was still lower than morphine, probably due to a reduced BBB penetration [175].

To further improve BBB permeability and plasma stability, the disulfide bridge in cyclic biphalin structure was substituted by a xylene bridge using CLIPS technology [176]. Novel biphalin analogs in which the two thiol groups of the d-Cys residues in position 2,2′ were linked by using different dibromoxylene regioisomers (*o*-dibromoxylene, *m*-dibromoxylene, and *p*-dibromoxylene) were synthesized. Among others, the analog containing the *o*-xylene moiety elicited strong antinociception in in vivo assays and was more potent than the biphalin after i.c.v., i.t., and s.c. administrations. A long-lasting antinociceptive effect after s.c. injection indicated improved human plasma stability and a better BBB penetration compared to biphalin [176].

**Figure 5:**
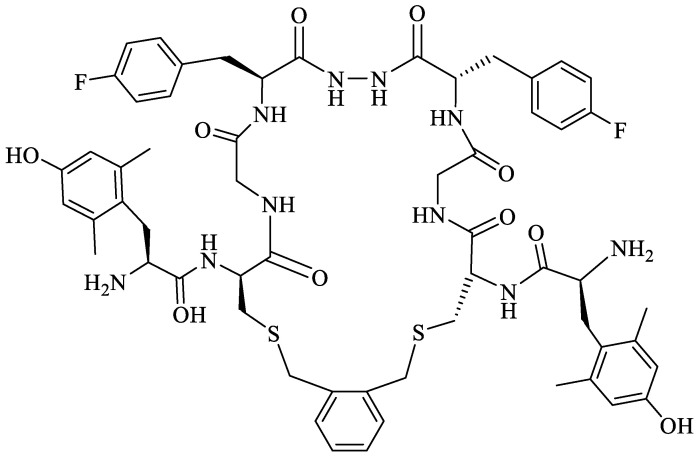
Structure of (H-Dmt-c[d-Cys-Gly-(pF)Phe])_2_-hydrazine-o-xylene (MACE4).

Based on the structure of one of the most potent linear biphalin analogs AM94 and cyclic biphalin with piperazine or hydrazine linker with or without a xylene bridge, Stefanucci et al., obtained novel fluorinated cyclic analogs of biphalin with excellent to a modest binding affinity for MOR, DOR and KOR [178]. The novel compounds incorporating xylene bridge and hydrazine linker (MACE2 and MACE4) (Figure 5) or piperazine linker (MACE3) exhibited a significantly long half-life with enhanced plasma human plasma stability and showed stronger antinociceptive effects after peripheral administration in comparison to biphalin and AM94 [178].

**Figure 6:**
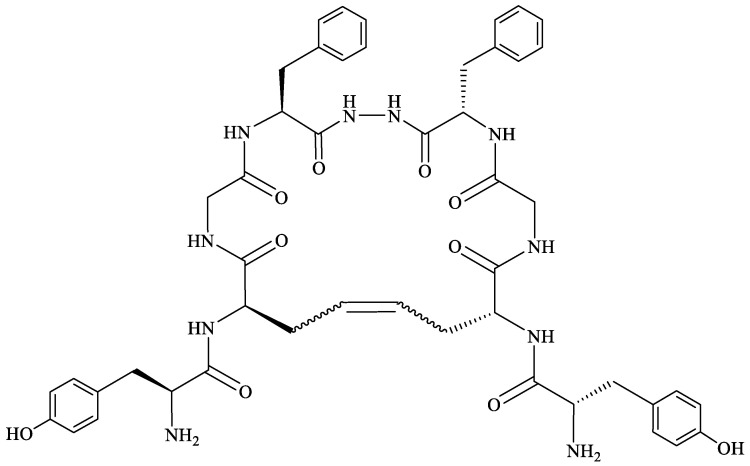
Structure of (H-Tyr-c[d-allyl-Gly-Gly-Phe])_2_-hydrazine (ABAM A: cis isomer, ABAM B: trans isomer).

In searching for more stable and versatile peptides with improved pharmacokinetic properties, replacing the labile S–S junction in cyclic peptides with an uncleavable C–C bond appears to be a promising strategy [179]. Using a ring-closing metathesis reaction, Stefanucci et al. [180] obtained two cis- and trans-cyclic olefin-bridged analogs of biphalin: ABAM-A and ABAM-B (Figure 6). The new ABAM compounds were MOR agonists/DOR partial antagonists, different from the mixed ΜOR/DOR agonist activity exerted by biphalin and its cyclic analogs reported so far in the literature. The analogs elicited a strong antinociceptive effect after i.c.v. and i.v. administration, higher than that of the previously described cyclic biphalin analogs containing a disulfide bridge between the side chains of two d-Cys or d-Pen residues [152,175].

### 3.4. Bifunctional Analogs: Hybrid Peptides

A promising approach for developing novel opioid analgesics with limited side effects is combining opioids with other neurotransmitters involved in pain perception (e.g., cholecystokinin, neurotensin, substance P, etc.). Such novel hybrids, also known as multitarget ligands that simultaneously target opioid and nonopioid receptors, can overcome the current limitations of single-target opioid analgesics [181,182,183,184].

In order to better understand the role of the neurokinin system in opioid-induced antinociception, Wtorek and co-workers synthesized a series of hybrid peptides combining previously described cyclic mixed MOR/KOR agonist C36 with either substance P (NK1 agonist) or spantide II (NK1 antagonist) fragments [185]. All obtained hybrids were characterized by high affinity to MOR and KOR, although lower compared to the opioid fragment. Two analogs, opioid agonist/NK1 antagonist H-Tyr-c[d-Lys-Phe-Phe-Asp]-Asn-d-Trp-Phe-d-Trp-Leu-Nle-NH_2_ and opioid agonist/NK1 agonist H-Tyr-c[d-Lys-Phe-Phe-Asp]-Gln-Phe-Phe-Gly-Leu-Met-NH_2_ that had high affinity to NK1 were selected for in vivo studies. In the writhing test in mice, both hybrids showed a significant antinociceptive effect and did not cause the development of tolerance or constipation, the typical side effects for opioids.

The MOR and NOP receptors are co-localized in the brain structures and share common signaling pathways. Recently, mixed MOR/NOP ligands came to the spotlight for their favorable functional profiles, circumventing the harmful effects of pure MOR agonists [186,187]. The first opioid agonist/nociceptin antagonist, H-Dmt-d-Arg-Aba-βAla-Arg-Tyr-Tyr-Arg-Ile-Lys-NH_2_ (where Aba = 4-amino-2-benzazepinone), was reported by Guillemyn and co-workers. When given i.v., this compound produced a potent antinociceptive effect in both acute and neuropathic pain models without inducing significant respiratory depression [188].

Wtorek et al., reported two novel chimeric peptides composed of MOR/KOR cyclic ligand C36 and the NOP receptor-binding peptide Ac-Arg-Tyr-Tyr-Arg-Ile-Lys-NH_2_, linked either directly (KW-495) or through a three-glycine spacer KW-496 [30]. In vitro binding and functional assays showed that KW-496 was a mixed ligand with inverted KOR > MOR affinity without activity at the NOP receptor. On the other hand, KW-495 activated the NOP receptor, although approximately 6-fold weaker than Ac-Arg-Tyr-Tyr-Arg-Ile-Lys-NH_2_, and was a mixed MOR/KOR/NOP agonist. Analog KW-495 produced a dose-dependent antinociceptive effect when given i.t., and this effect was antagonized by both the universal opioid receptor antagonist naloxone and the selective NOP receptor antagonist SB-612111, indicating the involvement of both classical opioid and NOP receptors in the mediation of its antinociceptive activity. The new chimeric peptide did not alter the mice’s locomotor activity, motor coordination, and balance.

The neuropeptide FF (NPFF) system plays an important role in regulating many physiological or pathological processes. Neuropeptide FF receptors 1 and 2 (NPFFR1 and NPFFR2) represent a relatively new target for many therapeutic applications, including modulation of opioid’s antinociception, tolerance, dependence, and other opioid side effects [189]. Recently, several linear multitarget peptide agonists of opioid/NPFF receptors have been reported [190]. These peptides produced potent, non-tolerance-forming antinociceptive activity with limited side effects. However, they were metabolically labile and exhibited poor BBB permeation [190]. Using disulfide-bond modification, Zhang et al., obtained two cyclic analogs H-Tyr-c[d-Cys-Gly-Phe-Cys]-Pro-Gln-Arg-Phe-NH_2_, (OFP006) and H-Tyr-c[d-Cys-Gly-NMe-Phe-Cys]-Pro-Gln-Arg-Phe-NH_2_ (OFP011) [190,191]. New cyclic disulfide analogs similar to their corresponding linear peptides functioned as multifunctional agonists for MOR, DOR, KOR-opioid, NPFF 1, and NPFF2 receptors in in vitro experiments, but they had improved BBB permeability, metabolic stability, and antinociceptive potency compared with their linear parent peptides [190,191]. The antinociceptive tolerance of analog OFP006 was greatly reduced after s.c. injection compared to fentanyl, as was the rewarding effect, withdrawal reaction, and gastrointestinal inhibition [191]. Behavioral experiments revealed that s.c. or oral (p.o.) administration of OFP011 resulted in potent and long-lasting antinociceptive activity in different pain models, with reduced opioid-like side effects, including constipation, tolerance, abuse potential, and respiratory depression [190]. On the other hand, the amide-bond cyclized peptide H-Tyr-c[d-Lys-Gly-NMe-Phe-Asp]-Pro-Gln-Arg-Phe-NH_2_ exhibited peripherally restricted antinociception [122]. These results suggest that the multifunctional opioid/NPFF receptor agonists are a promising strategy for the long-term treatment of moderate to severe nociceptive and pathological pain with fewer side effects. Recently, Drieu et al., obtained bifunctional peptididomimetic, H-Dmt-D-Arg-Aba-βAla-Bpa-Phe-NH_2_ (KGFF09) (Aba = 4-amino-2-benzazepinone, Bpa = 4-benzoyl-L-phenylalanine), possessing MOR agonist and NPFF receptor antagonist activity. KGFF09 displayed potent antinociception after subcutaneous (s.c.) administration in acute and chronic inflammatory pain in mice with a reduced propensity for unwanted side effects of conventional opioid analgesics, such as morphine, including respiratory depression, analgesic tolerance, opioid-induced hyperalgesia and physical dependence [192]. It was concluded that combining, within a single molecule, the G-protein-biased MOR agonism and NPFF receptor antagonism have beneficial effects on both acute and chronic side effects of classic opiates.

Further experiments revealed an effective and potent antinociceptive activity of KGFF09 also in a mouse model of visceral pain after s.c. injection with the absence of rewarding and locomotor dysfunction following chronic treatment [193].

It could be concluded that the development of dual MOR agonists/NPFF receptor agonists or antagonists paves the way for the design of potent and safer therapeutic treatments for various pain conditions.

### 3.5. Adding Novel Functionality: Peptide Conjugates

Among different strategies, conjugation with the oligoarginine vector, a kind of cationic cell-penetrating peptide, has been shown to enhance the permeation of the compounds through the BBB and their brain delivery [194]. To develop novel compounds with clinical potential, Zhang et al., designed and synthesized a series of endomorphin analogs by combined modifications, including cyclization, Dmt substitution in Tyr^1^, chlorination of Phe^4^ at the para-position, and C-terminal oligoarginine-vector conjugation [195]. The obtained conjugates were potent MOR agonists with enhanced stability and lipophilicity in comparison to endomorphins. Analogs H-Dmt-c(Cys-Trp/Phe-(pCl)Phe-Cys)-Gly-d-Arg-d-Arg-OH induced significant and prolonged antinociceptive effects in acute pain with reduced or no opioid-like side effects on gastrointestinal transit, conditioned place preference (CPP), and motor impairment after central and peripheral administration. New conjugates showed reduced acute antinociceptive tolerance, particularly nontolerance-forming antinociception at the peripheral level. In addition, they also produced long-acting antiallodynic effects against neuropathic and inflammatory pain [195].

**Figure 7:**
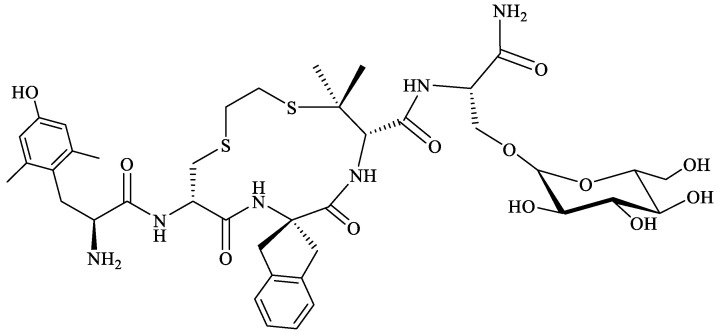
Structure of the peptide conjugate H-Dmt-c(SCH_2_CH_2_S)[d-Cys-Aci-d-Pen]-Ser(Glc)-NH_2_.

Starting from the structures of MOR-selective H-Tyr-c(SCH_2_CH_2_S)[d-Cys-Phe-d-Pen]-NH_2_ (JOM-6) [196] (Pen = β,β-dimethylcysteine) and DOR-selective H-Tyr-c[d-Cys-Phe-d-Pen]-OH (JOM-13) [197] cyclic tetrapeptides, Purington et al., synthesized, by a replacement of the Tyr and Phe residues in JOM-13 with a bulkier and more constrained Dmt and 2-aminoindane-2-carboxylic acid (Aci), respectively, a new cyclic analog H-Dmt-c(SCH_2_CH_2_S)[d-Cys-Aci-d-Pen]-OH (KSK-103) [198]. KSK-103 displayed MOR agonist/DOR antagonist activity and had a greater antinociceptive potency than morphine, but its main drawback was a poor bioavailability profile [198]. Glycosylation of this cyclopeptide by the addition of *C*-terminal β-glucosylserine residue [Ser(β-Glc)NH_2_] resulted in a cyclic analog, H-Dmt-c(SCH_2_CH_2_S)[d-Cys-Aci-d-Pen]-Ser(β-Glc)-NH_2_ (Figure 7), with increased bioavailability, that showed antinociceptive effect similar to morphine in the mouse tail-flicking model after i.p. administration, but did not cause acute tolerance [199].

The search for novel analogs with the potential to be good drug candidates with antinociceptive properties involves the selection of promising sequences, structure optimization by amino acid substitution, amide bond replacement, stabilizing conformation by cyclization of the molecule, and combining multiple structures for activity or physicochemical benefits [31,116]. The rational design of an opioid peptide takes into account metabolic stability and SAR, as well as pharmacokinetic and pharmacodynamic properties [31]. The biases and demands on specificity, reduction of possible adverse effects, and even the sustainable and environmentally friendly synthesis methods are being considered [200,201]. There is no silver bullet or single strategy to produce an ideal antinociceptive agent [200]. The search continues, with the limits and barriers being crossed and new challenges appearing.

## 4. Drug Delivery Systems for Antinociceptive Peptides

In general, clinical applications of opioid peptides have been limited due to their poor metabolic stability. Natural peptides undergo enzymatic degradation, making oral administration challenging or impossible [201]. The research is focused not only on more efficient antinociceptive activity but also on improved bioavailability and stability. The main strategies include the design of analogs resistant to peptide metabolism by introducing nonproteinaceous amino acids, cyclization, or peptide bond replacement. Another approach explores the concept of providing protection to the peptide by a delivery method, either based on non-oral administration, i.e., inhaled, buccal, intranasal, or transdermal, or employing molecular drug delivery systems [31,116,202]. With the opioid crisis and growing demand for pain relief, the idea of designing, adapting, and using drug delivery systems is gaining importance [203]. Another aspect of the involved research is the design aimed at extending the drug release profile of delivery systems to consider short-acting opioid (SAO) or long-acting opioid (LAO) formulations.

Several extended-release systems have been developed for opioid analgesic drugs [204,205] to produce stable drug concentration, limit the dosing frequency, reduce toxicity, and, in general, diminish side effects and improve patient’s quality of life [204]. However, peptide-based antinociceptive compounds are still at the experimental stage [204,206]. The main strategies employed in extended-release systems are based on encapsulation of the cargo into hydrogels, liposomes, and micelles, complexation and conjugation to dendrimers, and attachment to nanoparticles [207,208]. Macroscopic systems based on microneedles are intensively studied for transdermal delivery [209,210,211], whereas noninvasive nasal delivery gains attention, especially for peptide drug candidates, because of sidetracking metabolic traps and the proximity to CNS [212].

Hydrophilic compounds with limited stability create complications in drug delivery systems. In the case of antinociceptive peptides, dermorphin, and endomorphin-2 were selected for the study on the application of peptide hydrogels for controlled drug-delivery systems. The injectable amphipathic peptide hydrogels were investigated for morphine as well as opioid peptide cargoes [213]. Peptide-based hydrogels attract a lot of interest due to their biocompatibility and biodegradability [213,214].

Neurotoxin-1 (NT) was prepared for nasal delivery by loading on polymeric nanoparticles coated with polysorbate. The concentration of neurotoxin in the brain was higher after intranasal delivery of modified nanoparticles as compared with i.v. administration [215]. It is worth noting that solid lipid nanoparticles (SLN) were used to encapsulate nociceptin/orphanin FQ for reduction of airway hyperresponsiveness, which is not directly related to pain relief, although the results indicated improved bioavailability and delayed-release [216]. Intranasal delivery was also adopted for Opiorphin administration in the form of liposomal mucoadhesive thermo-sensitive gel containing PEGylated liposomal peptide dispersion [217].

The idea of using multimerized peptides for increased resistance to proteolytic enzymes was investigated for various applications in dendrimeric-like structures [218,219,220]. Dendrimers formed from one of conotoxin peptides, χ-MrIA, NGVCCGYKLCHOCa, were slightly more effective in vitro than the PEGylated analogs, although their potency was comparable to the parent peptide. However, in the rat pain model, the macromolecules were inactive, probably due to limited diffusion in the spinal cord after i.t. delivery [221]. Peptide welding technology synthesized bifunctional peptide constructs combining selective NOP receptor and MOR ligands as heterotetramers [222]. Dual NOP/MOR agonists present an improved profile with limited side effects and abuse risk [223]. Multiplied active peptides obtained in welding procedure, for example, H-PWT1-N/OFQ-[Dmt^1^]-dermorphin, combining nociceptin/orphanin and dermorphin analog, were as active as parent peptides but exhibited prolonged action [224].

## 5. Conclusions

The search for novel antinociceptive agents is focused on a better understanding of molecular mechanisms of pain and the adaptation of emerging bioactive agents to desired functionality through innovative methods of drug design [225]. The promising structures are discovered through investigation of natural products and using in silico methods [226]. The bioactive peptides from animal secretions offer an interesting starting point in the search for pain relief medicine based on the evolutionary adaptation to various receptors. The modification strategies described in this review are directed at increasing the stability of emerging compounds and the improvement of their pharmacodynamic profile. The use of peptide sequence as the origin of a new drug candidate benefits from the vast knowledge of peptide structure modification and the impressive toolbox available to peptide chemists, from non-proteinaceous amino acids and cyclization protocols to conjugation and multiplication in delivery systems. The clinical application of peptides is still limited; however, as the barrier for protein drugs was finally breached [227], the road to peptide drugs is now open.

## Figures and Tables

**Table 1:** Endogenous mammalian opioid peptides and their selectivities for the opioid receptors.

Endogenous Peptide	Amino Acid Sequence	Opioid Receptor Affinity
	Mammalian	
[Met]enkephalin	H-**Tyr-Gly-Gly-Phe**-Met-OH	DOR, MOR
[Leu]enkephalin	H-**Tyr-Gly-Gly-Phe**-Leu-OH	(DOR >> MOR)
dynorphin A	H-**Tyr-Gly-Gly-Phe**-Leu-Arg-Arg-Ile-Arg-Pro-Lys-Leu-Lys-Trp-Asp-Asn-Gln-OH	KOR, MOR, DOR
dynorphin B	H-**Tyr-Gly-Gly-Phe**-Leu-Arg-Arg-Gln-Phe-Lys-Val-Val-Thr-OH	(KOR >> MOR and DOR)
β-endorphin	H-**Tyr-Gly-Gly-Phe**-Met-Thr-Ser-Glu-Lys-Ser-Gln-Thr-Pro-Leu-Val-Thr-Leu-Phe-Lys-Asn-Ala-Ile-Ile-Lys-Asn-Ala-Tyr-Lys-Lys-Gly-Glu-OH	MOR, DOR
		(MOR = DOR)
endomorphin-1	H-Tyr-**Pro**-Trp-Phe-NH_2_	MOR
endomorphin-2	H-Tyr-**Pro**-Phe-Phe-NH_2_	
nociceptin/orphanin FQ (N/OFQ)	H-**Phe-Gly-Gly-Phe**-Thr-Gly-Ala-Arg-Lys-Ser-Ala-Arg-Lys-Leu-Ala-Asn-Gln-OH	NOP

**Table 2:** Selected examples of atypical opioid peptides.

Origin	Peptide	Amino Acid Sequence	Opioid Receptor Affinity
β-casein	bovine β-casomorphin(1–7)	H-Tyr-Pro-Phe-Pro-Gly-Pro-Ile-OH	MOR
	human β-casomorphin(1–7)	H-Tyr-Pro-Phe-Val-Glu-Pro-Ile-OH	MOR
	morphiceptin	H-Tyr-Pro-Phe-Pro-NH_2_	MOR
hemoglobin	hemorphin-4	H-Tyr-Pro-Trp-Thr-OH	MOR
amphibian skin *	dermorphin	H-Tyr-d-Ala-Phe-Gly-Tyr-Pro-Ser-NH_2_	MOR
	dermenkephalin	H-Tyr-d-Met-Phe-His-Leu-Met-Asp-NH_2_	DOR
	deltorphin I	H-Tyr-d-Ala-Phe-Asp-Val-Val-Gly-NH_2_	DOR
	deltorphin II	H-Tyr-d-Ala-Phe-Glu-Val-Val-Gly-NH_2_	DOR

* more examples of peptides of animal origin are presented in Section 2.

**Table 3:** Selected peptides from sea snails.

Peptide	Sequence	Organism	Ref.
α-conotoxin RgIA	G**CC**SDPR**C**RYR**C**R disulfide bonds: C^2^–C^8^, C^3^–C^12^	*Conus regius*	[46]
α-conopeptide Eu1.6	G**CC**SNPA**C**MLKNPNL**C**a disulfide bonds: C^2^–C^8^, C^3^–C^16^	*Conus eburneus*	[47]
ω-conotoxin MVIIA (SNX-111, ziconotide, or Prialt^®^)	**C**KGKGAK**C**SRLMYD**CC**TGS**C**RSGK**C**a disulfide bonds: C^1^–C^16^, C^8^–C^20^, C^15^–C^25^	*Conus magus*	[43]
ω-conotoxin GVIA (SNX-124)	**C**KSOGSS**C**SOTSYN**CC**RS**C**NOYTKR**C**Ya disulfide bonds: C^1^–C^16^, C^8^–C^19^, C^15^–C^26^	*Conus geographus*	[43]
CVIID ω-conotoxin	**C**KSKGAK**C**SKLMYD**CC**SGS**C**SGTVGR**C**a disulfide bonds: C^1^–C^16^, C^8^–C^20^, C^15^–C^27^	*Conus catus*	[43,48]
ω-conotoxin SO-3	**C**KAAGKP**C**SRIAYN**CC**TGS**C**RSGK**C**a disulfide bonds: C^1^–C^16^, C^8^–C^20^, C^15^–C^25^	*Conus striatus*	[43,44]
FVIA ω-conotoxin	**C**KGTGKS**C**SRIAYN**CC**TGS**C**RSGK**C**a disulfide bonds: C^1^–C^16^, C^8^–C^20^, C^15^–C^25^	*Conus fulmen*	[49]
CVIE ω-conotoxins	**C**KGKGAS**C**RRTSYD**CC**TGS**C**RSGR**C**a disulfide bonds: C^1^–C^16^, C^8^–C^20^, C^15^–C^25^	*Conus catus*	[50]
CVIF ω-conotoxins	**C**KGKGAS**C**RRTSYD**CC**TGS**C**RLGR**C**a disulfide bonds: C^1^–C^16^, C^8^–C^20^, C^15^–C^25^	*Conus catus*	[50]
MoVIA ω-conotoxins	**C**KPOGSK**C**SOSMRD**CC**TT**C**ISYTKR**C**RKYYN disulfide bonds: C^1^–C^16^, C^8^–C^19^, C^15^–C^26^	*Conus moncuri*	[45]
MoVIB ω-conotoxins	**C**KPOGSK**C**SOSMRD**CC**TT**C**ISYTKR**C**RKYY disulfide bonds: C^1^–C^16^, C^8^–C^19^, C^15^–C^26^	*Conus moncuri*	[45]
RsXXIVA	**C**KGQS**C**SSCSTKEFCLSKGSRLMYDCCTGSCCGVKTAGVT disulfide bonds: location not reported	*Conus regularis*	[51]

O represents hydroxyproline residue.

**Table 4:** Selected peptides from spider venom.

Peptide	Sequence	Organism	Ref.
Purotoxin-1 (PT1)	GY**C**AEKGIR**C**DDIH**CC**TGLK**C**K**C**NASGYN**C**V**C**RKKa disulfide bonds: C^3^–C^16^, C^10^–C^21^, C^15^–C^32^, C^23^–C^30^	*Lycosa kazakhstanicus*	[54]
Purotoxin-2 (PT2)	AKA**C**TPLLHD**C**SHDRHS**CC**RGDMFKYV**C**D**C**FYPEGEDKTEV**C**S**C**QQPKSHKIAEKIIDKAKTTLa disulfide bonds: C^4^–C^19^, C^11^–C^28^, C^18^–C^44^, C^30^–C^42^	*Lycosa kazakhstanicus*	[54]
PnTx2-6 (δ-CNTX-Pn2a)	ATCAGQDQPCKETCDCCGERGECVCGGPCICRQGYFWIAWYKLANCKK disulfide bonds: C^3^–C^17^, C^10^–C^23^, C^14^–C^46^, C^16^–C^31^, C^25^–C^29^	*Phoneutria nigriventer*	[55,56]
PnTx2-5	ATCAGQDQTCKVTCDCCGERGECVCGGPCICRQGNFLIAWYKLASCKK disulfide bonds: C^3^–C^17^, C^10^–C^23^, C^14^–C^46^, C^16^–C^31^, C^25^–C^29^	*Phoneutria nigriventer*	[55,56]
PnTx3-1	AE**C**AAVYER**C**GKGYKR**CC**EERP**C**K**C**NIVMDN**C**T**C**KKFISEL disulfide bonds: C^3^–C^18^, C^10^–C^23^, C^17^–C^34^, C^25^–C^32^	*Phoneutria nigriventer*	[56]
PnTx3-2 (Tx3-2, PNTx3-2)	A**C**AGLYKK**C**GKGASP**CC**EDRP**C**K**C**DLAMGN**C**I**C**KKKFIEFFGGGK disulfide bonds: C^2^–C^17^, C^9^–C^22^, C^16^–C^33^, C^24^–C^31^	*Phoneutria nigriventer*	[56]
PnTx3-3 (Phα1β)	G**C**ANAYKS**C**NGPHT**CC**WGYNGYKKA**C**I**C**SGXNWK disulfide bonds: C^2^–C^16^, C^9^–C^26^, C^15^–C^28^	*Phoneutria nigriventer*	[56,57]
PnTx3-6 (Phα1β)	A**C**IPRGEI**C**TDD**C**E**CC**GCDNQ**C**Y**C**PPGSSLGIFK**C**S**C**AHANKYF**C**NRKKEK**C**KKA disulfide bonds: C^2^–C^16^, C^9^–C^22^, C^13^–C^52^, C^15^–C^37^, C^18^–C^45^, C^24^–C^35^	*Phoneutria nigriventer*	[58]
PnTx3-4	SCSINVGDFCDGKKDDCQCCRDNAFCSCVIFGYKTNCRCEVGTTATSYGICMAKHKCGRQTTCTKPCLSKRCKKNHG disulfide bonds: C^2^–C^20^, C^10^–C^26^, C^17^–C^51^, C^19^–C^39^, C^28^–C^37^, C^57^–C^63^, C^67^–C^72^	*Phoneutria nigriventer*	[56]
PnTx3-5	G**C**IGRNES**C**KFDRHG**CC**WPWS**C**S**C**WNKEGQPESDVW**C**E**C**SLKIGK disulfide bonds: C^2^–C^17^, C^9^–C^22^, C^16^–C^39^, C^24^–C^37^	*Phoneutria nigriventer*	[56,59]
PnTx4(6-1) (δ-Ctenitoxin-Pn1a, δ-CNTX-Pn1a)	**C**GDINAA**C**KED**C**D**CC**GYTTA**C**D**C**YWSKS**C**K**C**REAAIVIYTAPKKKLT**C** disulfide bonds: C^1^–C^15^, C^8^–C^21^, C^12^–C^48^, C^14^–C^31^, C^23^–C^29^	*Phoneutria nigriventer*	[56,60]
β-TRTX-Cd1a	D**C**LGWFKS**C**DPKNDK**CC**KNYS**C**SRRDRW**C**KYDLa disulfide bonds: C^2^–C^17^, C^9^–C^22^, C^16^–C^29^	*Ceratogyrus darling*	[61]
JzTx-34	A**C**REWLGG**C**SKDAD**CC**AHLE**C**RKKWPYH**C**VWDWTV disulfide bonds: C^2^–C^16^, C^9^–C^21^, C^15^–C^29^	*Chilobrachys guangxiensis*	[62]
Hainantoxin-IV (μ-TRTX-Hhn1b, HNTX-IV)	E**C**LGFGKG**C**NPSNDQ**CC**KSSNLV**C**SRKHRW**C**KYEIa disulfide bonds: C^2^–C^17^, C^9^–C^24^, C^16^–C^31^	*Ornithoctonus hainana*	[63]
Huwentoxin-I (HWTX-I or HWAP-I)	A**C**KGVFDA**C**TPGKNE**CC**PNRV**C**SDKHKW**C**KWKL disulfide bonds: C^2^–C^17^, C^9^–C^22^, C^16^–C^29^	*Ornithoctonus huwena*	[64,65]
Huwentoxin-XVI (HWTX-XVI)	**C**IGEGVP**C**DENDPR**CC**SGLV**C**LKPTLHGIWYKSYY**C**YKK disulfide bonds: C^1^–C^16^, C^8^–C^21^, C^15^–C^36^	*Ornithoctonus huwena*	[66]
SNX-482	GVDKAG**C**RYMFGG**C**SVNDD**CC**PRLG**C**HSLFSY**C**AWDLTFSD disulfide bonds: C^7^–C^21^, C^14^–C^26^, C^20^–C^33^	*Hysterocrates gigas*	[67]
Phlotoxin 1 (Ph1Tx1)	A**C**LGQWDS**C**DPKASK**CC**PNYA**C**EWKYPWCRYKLF disulfide bonds: C^2^–C^17^, C^9^–C^22^, C^16^–C^29^	*Phlogiellus spider*	[68]
ω-Agatoxin IVA	KKK**C**IAKDYGR**C**KWGGTP**CC**RGRG**C**I**C**SIMGTN**C**E**C**KPRLIMEGLGLA disulfide bonds: C^4^–C^20^, C^12^–C^25^, C^19^–C^36^, C^27^–C^34^	*Agelenopsis aperta*	[69]
Tick peptide	LVVYPWTKM	*Amblyomma testindiarium* (tick)	[70]

**Table 5:** Selected peptides from scorpions.

Peptide	Sequence	Organism	Ref.
BmK AGAP	VRDGYIADDKN**C**AYF**C**GRNAY**C**DDE**C**KKNGAESGY**C**QWAGVYGNACW**C**YKLPDKVPIRVPGK**C**NGG disulfide bonds; C^12^–C^63^, C^16^–C^36^, C^22^–C^46^, C^26^–C^48^	*Buthus martensi*	[80]
BmK AGAP-SYPU2	VKDGYIVDDKN**C**AYF**C**GRNAY**C**DDE**C**EKNGAESGY**C**QWAGVYGNA**C**W**C**YKLPDKVPIRVPGR**C**NG disulfide bonds: C^12^–C^63^, C^16^–C^36^, C^22^–C^46^, C^26^–C^48^	*Buthus martensi*	[80]
BmK AS	DNGYLLDKYTGCKVWCVINNESCNSECKIRGGYYGYCYFWKLACFCQGARKSELWNYNTNKCDGKL disulfide bonds: C^12^–C^62^, C^16^–C^37^, C^23^–C^44^, C^27^–C^46^	*Buthus martensi*	[78,81]
BmK AS1	DNGYLLNKYTGCKIWCVINNESCNSECKLRRGNYGYCYFWKLACYCEGAPKSELWAYETNKCDGKL disulfide bonds: C^12^–C^62^, C^16^–C^37^, C^23^–C^44^, C^27^–C^46^	*Buthus martensi*	[81]
BmK IT2	DGYIKGKSG**C**RVA**C**LIGNQG**C**LKD**C**RAYGASYGY**C**WTWGLA**C**W**C**EGLPDNKTWKSESNT**C**G disulfide bonds: C^10^–C^60^, C^14^–C^35^, C^21^–C^42^, C^25^–C^44^	*Buthus marten*	[78,82]
BmK-YA	YGGYMNPAa	*Buthus marten*	[83]
BmK Ang P1	KKNGYAVDSSGKVAE	*Buthus marten*	[84]
TsNTxP	MKRMILFISCLLLIDIVVGGREGYPADSKGCKITCFLTAAGYCNTECTLKKGSSGYCAWPACYCYGLPDSVKIWTSETNKCGKK disulfide bonds: C^31^–C^81^, C^35^–C^57^, C^43^–C^62^, C^47^–C^64^	*Tityus serrulatus*	[85]
Hetlaxin	IS**C**TGSKQ**C**YDP**C**KKKTG**C**PNAK**C**MNKS**C**X**C**YG**C**a disulfide bonds: C^3^–C^24^, C^9^–C^29^, C^13^–C^31^, C^19^–C^34^	*Heterometrus laoticus*	[86]

**Table 6:** Selected peptides from other arthropods.

Peptide	Sequence	Organism	Ref.
[Thr^6^]-bradykinin (Thr^6^-BK)	RPPGFTPFR	*Polybia occidentalis* (wasp)	[93]
Pallipin-III	SIKKHKCIALLKRRGGSKLPFCa	*Agelaia pallipes pallipes* (wasp)	[94]
Protonectin	ILGTILGLLKGLa	*Parachartergus fraternus* (wasp)	[95]
Agelaia-MP I	INWLKLGKAIIDALa	*Parachartergus fraternus* (wasp)	[94]
Melittin	GIGAVLKVLTTGLPALISWIKRKRQQ	*Apis mellifera* (bee)	[96]
Proctolin	RYLPT	*Perplaneta americana* (cockroach)	[87]
*Neb*-TMOF	NPTNLH	*Neobellieria bullata* (fly)	[89]
Alloferon	HGVSGHGQHGVHG	*Calliphora vicina* (fly)	[90]
LPK	pETSFTPRLa	*Leucophaea madera* (cockroach)	[88]
MAS MT I	pEDVVHSFLRFa	*Manduca sexta* (cockroach)	[91]
Poneratoxin	FLPLLILGSLLMTPPVIQAIHDAQRa	*Paraponera clavata* (ant)	[92]

pE represents pyroglutamic acid residue.

**Table 7:** Selected peptides from amphibia.

Peptide	Sequence	Organism	Ref.
Dermorphin	H-Tyr-d-Ala-Phe-Gly-Tyr-Pro-Ser-NH_2_	*Phyllomedusa sauvagei*	[100]
[Hyp^6^]-dermorphin	H-Tyr-d-Ala-Phe-Gly-Tyr-Hyp-Ser-NH_2_	*Phyllomedusa sauvagei*	[105]
[Lys^7^]-dermorphin-OH	H-Tyr-d-Ala-Phe-Gly-Tyr-Pro-Lys-OH	*Phyllomedusa bicolor*	[101]
[Lys^7^]-dermorphin-NH_2_	H-Tyr-d-Ala-Phe-Gly-Tyr-Pro-Lys-NH_2_	*Phyllomedusa bicolor*	[101]
[Trp^4^-NH_2_]-dermorphin	H-Tyr-d-Ala-Phe-Trp-Tyr-Pro-Ser-NH_2_	*Phyllomedusa bicolor*	[101]
[Trp^4^, Asn^7^]-dermorphin	H-Tyr-d-Ala-Phe-Trp-Tyr-Pro-Asn-OH	*Phyllomedusa bicolor*	[105]
[Trp^4^, Asn^7^-NH_2_]-dermorphin	H-Tyr-d-Ala-Phe-Trp-Tyr-Pro-Asn-NH_2_	*Phyllomedusa bicolor*	[101]
[Trp^4^, Asn-OH^5^]-dermorphin	H-Tyr-d-Ala-Phe-Trp-Asn-OH	*Phyllomedusa bicolor*	[101]
Y10A	H-Tyr-d-Ala-Phe-Gly-Tyr-Pro-Ser-Gly-Glu-Ala-OH	*Phyllomedusa sauvagei*	[101]
Y131	H-Tyr-d-Ala-Phe-Gly-Tyr-Pro-Ser-Gly-Glu-Ala-Lys-Lys-Ile-OH	*Phyllomedusa sauvagei*	[101]
DAla-deltorphin I	H-Tyr-d-Ala-Phe-Asp-Val-Val-Gly-NH_2_	*Phyllomedusa bicolor*	[105]
DAla-deltorphin II	H-Tyr-d-Ala-Phe-Glu-Val-Val-Gly-NH_2_	*Phyllomedusa bicolor*	[105]
DMet-deltorphin, dermenkephalin, Deltrophin	H-Tyr-d-Met-Phe-His-Leu-Met-Asp-NH_2_	*Phyllomedusa bicolor*	[105]
Deltorphin	H-Tyr-Ala-Phe-Gly-Tyr-Pro-Ser-NH_2_	*Phasmahyla jandaia*	[101]
Dlle-deltorphin	H-Tyr-d-Ile-Phe-His-Leu-Met-Asp-NH_2_	*Pachymedusa dacnicolor*, *Agalychnis annae*	[101]
[Met(Ox)]^6^-deltorphin	H-Tyr-Met-Phe-His-Leu-Met(O)-Asp-NH_2_	*Phasmahyla jandaia*	[101]
DLeu-deltorphin-17	H-Tyr-d-Leu-Phe-Ala-Asp-Val-Ala-Ser-Thr-Ile-Gly-Asp-Phe-Phe-His-Ser-Ile-NH_2_	*Phasmahyla jandaia*	[101]
Odorranaopin	H-Asp-Tyr-Thr-Ile-Arg-Thr-Arg-Leu-His-Gln-Glu-Ser-Ser-Arg-Lys-Val-Leu-OH	*Odorrana graham*	[104]
Tryptophyllin L1.2	H-Phe-Pro-Trp-Leu-NH_2_	*Litoria rubella*	[101]
Tryptophyllin L3.1	H-Phe-Pro-Trp-Pro-NH_2_	*Litoria rubella*	[101]
-	H-Phe-Pro-Kyn-Leu-NH_2_	*Litoria rubella*	[101,106]
Adenoregulin	GLWSKIKEVGKEAAKAAAKAAGKAALGAVSEAV	*Phyllomedusa bicolor*	[101]
Tt7	EQDPKCLLPRNLGKGKGSTIRYYYDKSAGT disulfide bonds: C^6^–C^4^, C^2^–C^3^	*Trachycephalus typhonius*	[107]

Kyn-kynurenine. For clarity, the short sequences in this table are presented in a three-letter code to accommodate nonproteinaceous amino acid residues and to avoid confusion between D-Ala (frequently indicated as a) and C-terminal amide.

**Table 8:** Selected peptide from snake venom.

Peptide	Sequence	Organism	Ref.
Mambalgin-1	LK**C**YQHGKVVT**C**HRDMKF**C**YHNTGMPFRNLKLILQG**C**SSS**C**SETENNK**CC**STDR**C**NK disulfide bonds: C^3^–C^19^, C^12^–C^37^, C^41^–C^49^, C^50^–C^55^	*Dendroaspis polylepis*	[108]
Mambalgin-2	LK**C**FQHGKVVT**C**HRDMKF**C**YHNTGMPFRNLKLILQG**C**SSS**C**SETENNK**CC**STDR**C**NK disulfide bonds: C^3^–C^19^, C^12^–C^37^, C^41^–C^49^, C^50^–C^55^	*Dendroaspis polylepis*	[108]
Mambalgin-3	LK**C**YQHGKVVTCHRDMKF**C**YHNIGMPFRNLKLILQG**C**SSS**C**SETENNK**CC**STDR**C**NK disulfide bonds: C^3^–C^19^, C^12^–C^37^, C^41^–C^49^, C^50^–C^55^	*Dendroaspis angusticeps*	[108]
α-cobratoxin (α-CbTX, α-elapitoxin–Nk2a)	IR**C**FITPDITSKD**C**PNGHV**C**YTKTW**C**DAFCSIRGKRVDLG**C**AAT**C**PTVKTGVDIQ**C**CSTDN**C**NPFPTRKRP disulfide bonds: C^3^–C^20^, C^14^–C^41^, C^26^–C^30^, C^45^–C^56^, C^57^–C^62^	*Naja naja kaouthia*	[48,109]
Crotalphine	pEFSPEN**C**QGESQP**C** disulfide bond: C^7^–C^14^	*Crotalus durissus terrificus*	[110]
μ-EPTX-Na1a	LK**C**HNTQLPFIYKT**C**PEGKNL**C**FKATLKKFPLKFPKRG**C**ADN**C**PKNSALLKYV**CC**STDK**C**N disulfide bonds: C^3^–C^22^, C^15^–C^40^, C^44^–C^55^, C^56^–C^61^	*Naja atra*	[111]

**Table 9:** Common modifications of the opioid peptides.

Type of Modification	Peptidomimetic	Ref.
d-amino acids	[d-Ala^2^, d-Leu^5^]-Enkephalin (DADLE) 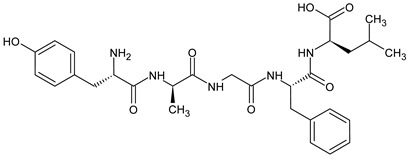	[124]
	[d-Ser^2^, d-Leu^5^, Thr^6^]-Enkephalin (DSLET) 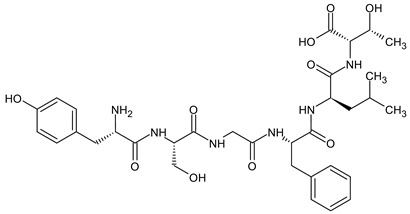	[128,129]
	[d-Ala^3^]-Dynorphin A-(1-11)-NH_2_ H-Tyr-Gly-d-Ala-Phe-Leu-Arg-Arg-Ile-Arg-Pro-Lys-NH_2_	[130]
β amino acids Homo-amino acids	(H-Tyr-d-Ala-Phe-Gly-β3-homo-Tyr)_2_hydrazine 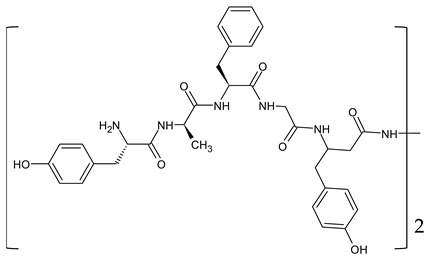	[131]
2′,6′-dimethyltyrosine (Dmt) derivatives	H-Dmt^1^-βPro^2^-Trp^3^-(2-furyl)Map^4^ -Endomorphin 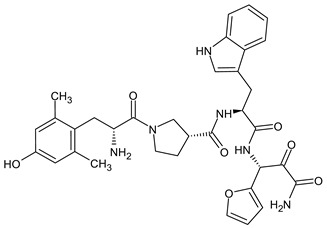	[132]
	H-Dmt-d-Arg-Phe-Lys-NH_2_ ([Dmt^1^]DALDA) 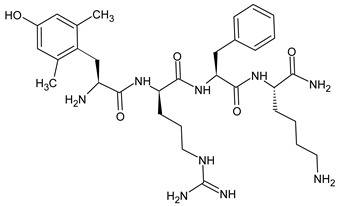	[133,134]
*N*- and *C*-terminal modifications	[d-Ala^2^,N-MePhe^4^,Gly-ol]-Enkephalin (DAMGO) 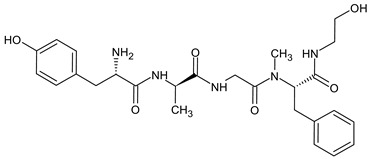	[127]
	guanidyl-Tyr-d-Ala-Gly-Phe-Leu-tetrazole 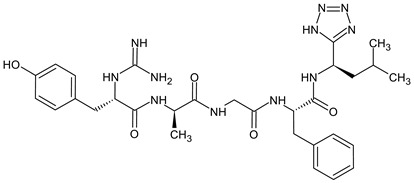	[126]
Replacement of the peptide bond	H-Tyr-d-Ala-Phe-Leu-Arg ψ (CH_2_NH) Arg-NH_2_ 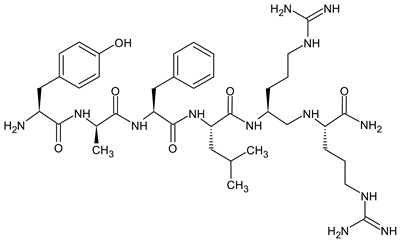	[135]

## Data Availability

No new data were created or analyzed in this study. Data sharing is not applicable to this article.
